# The Influence of Sample Size on Parameter Estimates in Three-Level Random-Effects Models

**DOI:** 10.3389/fpsyg.2019.01067

**Published:** 2019-05-21

**Authors:** Denise Kerkhoff, Fridtjof W. Nussbeck

**Affiliations:** ^1^Department of Psychology, Bielefeld University, Bielefeld, Germany; ^2^Department of Psychology, University of Konstanz, Konstanz, Germany

**Keywords:** random effects model, sample size, power analysis, three-level model, parameter estimation

## Abstract

In educational psychology, observational units are oftentimes nested within superordinate groups. Researchers need to account for hierarchy in the data by means of multilevel modeling, but especially in three-level longitudinal models, it is often unclear which sample size is necessary for reliable parameter estimation. To address this question, we generated a population dataset based on a study in the field of educational psychology, consisting of 3000 classrooms (level-3) with 55000 students (level-2) measured at 5 occasions (level-1), including predictors on each level and interaction effects. Drawing from this data, we realized 1000 random samples each for various sample and missing value conditions and compared analysis results with the true population parameters. We found that sampling at least 15 level-2 units each in 35 level-3 units results in unbiased fixed effects estimates, whereas higher-level random effects variance estimates require larger samples. Overall, increasing the level-2 sample size most strongly improves estimation soundness. We further discuss how data characteristics influence parameter estimation and provide specific sample size recommendations.

## Introduction

In educational research and the field of psychology in general, researchers oftentimes face the statistical problem of nested data structures. Such structures occur if measured entities belong to superordinate groups. For example, researchers might examine children (lowest level) nested within classrooms (medium level) and schools (highest level). In statistical models, this nested structure has to be respected. One prominent approach is multilevel analysis (MLA). The fundamental assumption of MLA is “that there is a hierarchical data set, […] with one single outcome or response variable […], and explanatory variables at all existing levels” (Hox et al., 2017, p. 8). Hence, a system of regression equations at different hierarchical levels describes the influence of the explanatory variables. MLA is also useful for repeated measures of the same variable over a period of time within the same entities (e.g., diary studies).

In general, disregarding hierarchical data structures leads to biased fixed effects estimates and standard errors (McNeish and Wentzel, 2017), which in turn influence the accuracy of significance tests (e.g., Chen, 2012) and statistical power calculations (e.g., Konstantopoulos, 2008). It can also lead to incorrect conclusions: the ecological fallacy describes the incorrect inference that effects at the group level are the same for individual members of the group (Piantadosi et al., 1988). The atomistic fallacy describes the opposite case, where conclusions based on results for a subgroup are erroneously generalized (e.g., Hox, 1995). Applying MLA reduces the chances of drawing these false conclusions based on inaccurate analysis results.

Besides these general advantages of MLA, the quality of estimation results in multilevel models is influenced by different characteristics (e.g., the number of units at the different levels, scale-level of variables, presence or absence of interaction effects, violation of assumptions, or estimator properties). Comprehensive analytical solutions to evaluate specific estimation results are, however, rare. In the past years, authors have derived formulae for power or sample size calculations for different three-level models (Heo and Leon, 2008; Konstantopoulos, 2008; Cunningham and Johnson, 2016; Hooper et al., 2016), but in comparison, empirical power has been found to be slightly lower than theoretical power (Heo and Leon, 2008). As an alternative to analytical solutions, simulation studies examine the estimation quality under specific sampling and data conditions, especially varying sample sizes. While the majority of such studies is concerned with two-level models (e.g., Maas and Hox, 2004, 2005), there are many research settings where multiple nesting structures frequently result in at least three-level data, such as the Program for International Student Assessment (PISA) or the Trends in International Mathematics and Science Study (TIMSS), which have been analyzed using two-level (Caponera and Losito, 2016) and three-level models (Webster and Fisher, 2000; Lamb and Fullarton, 2002). Alternatively, complex data structures easily require three-level MLA if a longitudinal component is added, such as schools (level 3) with students (level 2), which are studied on different measurement occasions (level 1). Notably, a distinction is to be made between the three-level nature of a dataset and the appropriate statistical model. Longitudinal dyadic data with distinguishable members, where members (level-2) of dyads (level-3) are measured over time (level-1), for example, have to be modeled as two-level data rather than three-level data (Laurenceau and Bolger, 2005; Kenny and Kashy, 2011; Planalp et al., 2017).

With the present simulation study, we highlight and address specific issues researchers in educational psychology or a setting with comparable samples, models, and effects face when considering MLA for three-level data. Specifically, we simulate and analyze prototypical data based on results of an empirical study in the field of educational psychology as a starting point to address the following questions: What is the overall required sample size and what is the optimal allocation of observational units across levels in order to obtain valid and reliable results? In particular, this study utilizes practice-relevant effects by generating nested data based upon the prototypical three-level regression model from an empirical study. Estimation quality of fixed effects and random effects variances (*r.e. variances* in the remainder) is assessed and compared by simulating various sample size conditions with different numbers of units at each data level, and analyzing these samples using MLA. By using data reflecting typical MLA-results and sample characteristics of an illustrative psychological study, these analyses are a practical approach to identify important factors in similar research settings when deciding on the appropriate overall sample size and allocation at the different levels.

## The Multilevel Model

Formulae (1) to (3) below provide the notation for the most general three-level regression model with one predictor per level as well as cross-level interactions, random slopes, and intercepts. Depending on the data, the hypothesized effects, and their interactions, models may in practice contain less (e.g., no random effects) or more (e.g., additionally predictors) components on each level. Given *k* = 1,…,K level-3 groups, *j* = 1,…,J level-2 subgroups, and *i* = 1,…,n level-1 units, this model contains the dependent variable *Y_ijk_*, and predictors X*_ijk_*, Z*_jk_*, and W*_k_*.

Level 1

(1) $$Y_{ijk}=\beta_{0jk}+\beta_{1jk}X_{ijk}+e_{ijk}\,$$

Level 2

(2) $$\begin{array}{c} \beta_{0jk}=\gamma_{00k}+\gamma_{01k}Z_{jk}+u_{0jk}\\ \beta_{1jk}=\gamma_{10k}+\gamma_{11k}Z_{jk}+u_{1jk} \end{array}$$

Level 3

(3) $$\begin{array}{c} \gamma_{00k}=\delta_{000}+\delta_{001}W_{k}+v_{00k}\\ \gamma_{10k}=\delta_{100}+\delta_{101}W_{k}+v_{10k}\\ \gamma_{01k}=\delta_{010}+\delta_{011}W_{k}+v_{01k}\\ \gamma_{11k}=\delta_{110}+\delta_{111}W_{k}+v_{11k} \end{array}$$

Regression equation (1) corresponds to the first level of the ML-model, with intercept β_0*jk*_, explanatory variable *X_ijk_* multiplied with the regression coefficient β_1*jk*_, and residuals *e_ijk_*. Its components are composed of subsequent higher-level equations. The next two formulae represent the second data level. The first part of (2) depicts how the intercept β_0*jk*_ is decomposed into the level-2 intercept γ_00_*_k_*, slope γ_01*k*_ times explanatory variable *Z_jk_*, and level-2 residual *u*_0*jk*_. The second part shows how the level-1 slope β_1*jk*_ is decomposed into the level-2 intercept γ_10*k*_, slope γ_11*k*_ times *Z_jk_*, and the residual *u*_1*jk*_. Analogously, formula (3) builds the third data level with level-3 predictor *W_k_*, relative intercepts δ_000_, δ_100_, δ_010_, and δ_110_, slopes δ_001_, δ_101_, δ_011_, and δ_111_, and residuals *v*_00*k*_, *v*_10*k*_, *v*_01*k*_, and *v*_11*k*_.

While intercepts (β_0*jk*_, γ_00*k*_, γ_10*k*_, δ_000_, δ_100_, δ_010_, δ_110_) and regression weights (β_1*jk*_, γ_01*k*_, γ_11*k*_, δ_001_, δ_101_, δ_011_, δ_111_) are the fixed effects of the model, the residuals *e_ijk_*, *u*_0*jk*_, *u*_1*jk*_, *v*_00*k*_, *v*_10*k*_, *v*_01*k*_, and *v*_11*k*_ are random effects. Their variances are estimated and denoted by ε^2^*_e_* (level-1 residual variance), τ^2^_*u*0_ (level-2 intercept variance), τ^2^_*u1*_ (level-2 slope variance), σ^2^_*v*0_ (level-3 intercept variance), and σ^2^_*v*1_ (level-3 slope variance). The higher level r.e. variances express the unexplained variability of intercept and slope parameters at the different levels, while ε^2^_*e*_ expresses the remaining residual variance. Hox et al. (2017), Chapter 2 provide an in-depth explanation of the function of each component for two-level models.

The dependency in the data resulting from such a multilevel structure is measured by the intraclass correlation coefficient (ICC), indicating how strongly units of the same cluster are more similar to each other than units of different clusters. The ICC values express the variability on one data level in relation to the overall variability in the data, and are hence calculated as in (4) to (6), with ε^2^*_e_* (level-1 variance), τ^2^_*u*0_ (level-2 intercept variance), and σ^2^_*v*0_ (level-3 intercept variance) of the unconditional model (i.e., a model including no predictor variables).

(4) $$\rho level\text{ 3}=\frac{\sigma_{v_{0}}^{2}}{\tau_{u_{0}}^{2}+\sigma_{v_{0}}^{2}+\varepsilon_{e}^{2}}$$

(5) $$\rho level\text{ 2}\left(a\right)=\frac{\tau_{u_{0}}^{2}+\sigma_{v_{0}}^{2}}{\tau_{u_{0}}^{2}+\sigma_{v_{0}}^{2}+\varepsilon_{e}^{2}},\text{or alternatively}$$

(6) $$\rho level\text{ 2}\left(b\right)=\frac{\tau_{u_{0}}^{2}}{\tau_{u_{0}}^{2}+\sigma_{v_{0}}^{2}+\varepsilon_{e}^{2}}$$

(5) and (6) both present versions for the level-2 ICC, with (5) including the level-3 r.e. variance component as additional variability. As Hox et al. (2017, p. 21) argue, both equations can be used to evaluate dependencies in the data: While (5) evaluates the correlation between two level-1 units of the same level-2 unit, (6) is advantageous if the aim is to measure the proportion of the total unexplained variance that is situated at level-2.

The MLA approach to clustered data is very flexible, yet these complex models require an adequate amount of observations at each level in order to obtain sound estimates of fixed effects and r.e. variances. In the remainder, we will first present coefficients representing the soundness of model-parameter estimates, review the literature on sample-size requirements in two- and three-level models, and finally describe the simulation study in detail. Throughout, we refer to the number of units on each level as follows: *N*_3_ = level-3 sample size, *N*_2_ = level-2 sample size, and *N*_1_ = level-1 sample size or number of measurement occasions in longitudinal assessments.

## Assessing Quality of Parameter Estimation

A common measure for assessing the accuracy of parameter estimates is the parameter estimation bias (*peb*) which calculates the fraction of under- or overestimation of a true value θ by its estimates θ*_k_*^∗^ in *n* samples using formula (7) (adapted from Bandalos and Gagné, 2014). Muthén and Muthén (2002) suggest that the bias should not exceed 0.10.

(7) $$peb=\frac{\Sigma_{k=1}^{n}\left(\frac{\theta_{k}*-\theta }{\theta }\right)}{n}$$

The *confidence interval* is commonly used to assess the accuracy of the estimation. The specific formula used to calculate the confidence interval depends on the estimation method and the (assumed) distribution of the parameter. Equation (8) expresses the general formula for regression coefficients with estimate θ^∗^ and its standard error *s*_θ_, assuming that the standard normal distribution with α/2-quantile *z*_α/2_ can be used for a specified type I error probability α (Davidson and MacKinnon, 1999):

(8) $$C_{\left(1-\alpha \right)}=\left[\theta^{*}+s_{\theta }z_{\alpha /2},\theta^{*}-s_{\theta }z_{\alpha /2}\right]$$

The *coverage* calculates the percentage of confidence intervals across analysis runs, which contain the true parameter. If the calculated coverage is lower (higher) than the proposed level, statistical tests will be conservative (liberal). For a proposed confidence level of 95%, Muthén and Muthén (2002) recommend a coverage rate between 91 and 98%.

Statistical power is “the likelihood that a researcher will be able to reject a specific null hypothesis when it is in fact false” (Murphy, 2011, p. 1082). In simulation studies with one sample generated and analyzed in each run, estimated power for a specific effect equals the percentage of runs yielding statistically significant estimates of this effect. As an alternative, the percentage of confidence intervals for this effect *excluding* zero can be used as a measure to indicate a significant effect. A power of at least 80% is regarded as acceptable for significance testing (e.g., Muthén and Muthén, 2002).

## Research on Sample Size in Multilevel Models

Research on sample size requirements mainly focus on two-level models, with fewer studies investigating general three-level models. Therefore, we outline main findings with respect to two- and three-level models. Tables 1–4 summarize the simulation conditions or parameters under investigation (two-level models: Table 1; three-level models: Table 2) and findings and recommendations (two-level models: Table 3; three-level models: Table 4) of previous studies.

**Table 1:** Investigated parameters of studies examining the impact of sample sizes on estimation results in two-level models.

Authors (Year)		Simulation conditions: Model/data, Sample sizes, and Parameters	Other conditions
Du and Wang (2016)	M	*X*_1*j*_∼*N*(0,1), *X*_2*j*_ dyad, *Z*_1_ dic., *X*_1*j*_^∗^*X*_2*j*_, *X*_2*j*_^∗^*Z*_1_, *X*_1*j*_^∗^*Z*_1__,_ *X*_2*j*_^∗^*X*_1*j*_^∗^*Z*1, *e_ij_*, *u*_0*j*_	Missing values (singletons): 0%, 10%, 30%, 50%, MCAR and MAR
	S	*N*_2_: 30, 50, 100, 150, 200, 300, 400, 500, 500, *N*_1_: 2	
	P	ICC: 0.1, 0.2, 0.3, 0.5, 0.7, ε^2^*_e_* = 0.5, γ_00_ = 1, other fixed ES = 0.3	
Spain et al. (2012)	M	Logistic model, *X*_1*j*_ dyad, *X*_2*j*_-X_5*j*_ cont., *e_ij_*, *u*_0*j*_	Estimation: generalized linear mixed model and hierarchical linear model
	S	*N*_2_: 50, 100, 250, 500, 2200, *N*_1_: 2	
	P	γ_00_ = -2.47, τ^2^_*u*0_ = 0.43, *X*_1*j*_ = 0.05, *X*_2*j*_ = 0.03, *X*_3*j*_ = -0.05, *X*_4*j*_ = 0.19, *X*_5*j*_ = 0.08	
Maas and Hox (2004)	M	*X*_1*j*_∼*N*(0,1), *Z*_1_∼*N*(0,1), *X*_1*j*_^∗^*Z*_1_, *e_ij_*, *u*_0*j*_, *u*_1*j*_	*u*_0*j*_, *u*_1*j*_ distribution: normal and non-normal (χ^2^_1_) estimator: ML and MLR
	S	*N*_2_: 30, 50, 100; *N*_1_: 5, 30, 50	
	P	ICC: 0.1, 0.2, 0.3, ε^2^*_e_* = 0.5, τ^2^_*u*0_ = τ^2^_*u*1_, γ_00_ = 1, other fixed ES = 0.3	
Maas and Hox (2005)	M	*X*_1*j*_∼*N*(0,1), *Z*_1_∼*N*(0,1), *X*_1*j*_^∗^*Z*_1_, *e_ij_*, *u*_0*j*_, *u*_1*j*_	–
	S	*N*_2_: 30, 50, 100; *N*_1_: 5, 30, 50	
	P	ICC: 0.1, 0.2, 0.3, ε^2^*_e_* = 0.5, τ^2^_*u*0_ = τ^2^_*u*1_, γ_00_ = 1, other fixed ES = 0.3	
Moineddin et al. (2007)	M	Logistic model, *X*_1*j*_∼*N*(0,1), *Z*_1_∼*N*(0,1), *X*_1*j*_^∗^*Z*_1_, *e_ij_*, *u*_0*j*_, *u*_1*j*_	–
	S	*N*_2_: 30, 50, 100; *N*_1_: 5, 30, 50	
	P	ICC: 0.04, 0.17, 0.38, τ^2^_*u*0_ = 0.13, 0.67, 2.0, γ_00_ = -1, other fixed ES = 0.3	
Mathieu et al. (2012)	M	*X*_1*j*_∼*N*(0,varying), *Z*_1_∼*N*(0,1), *X*_1*j*_^∗^*Z*_1_, *e_ij_*, *u*_0*j*_, *u*_1*j*_	Variable reliability:
	S	*N*_2_: 20, 40, 60, 115, *N*_1_: 3, 5, 7, 18	0.8, 0.9, 1.0
	P	ICC: 0.15, 0.30, ε^2^*_e_* = 1, various fixed effect sizes (ES = 0 to ES = 0.75)	level 1 slopes *SD*: 0.10, 0.17, 0.22


*Outcome continuous if not otherwise stated. *N*_*1*_, level-1 sample size per level-2 unit; *N*_*2*_, level-2 sample size. ICC, intraclass correlation coefficient. ES, effect size. SD, standard deviation. ML, maximum likelihood. MLR, maximum likelihood with robust standard errors. MAR, missing at random. dic, dichotomous. dyad, dichotomous dyad member indicator variable. *X_*ij*_, Z_*j*_, e_*ij*_, u_*0j*_, u_*1j*_, ε^*2*^_*e*_, γ_*00*_, τ^*2*^_*u0*_, τ^*2*^_*u1*_* refer to variables and effects on their respective level according to formulae (1) and (2) with indices adjusted for two-level models.*

**Table 2:** Investigated parameters of studies examining the impact of sample sizes on estimation results in three-level models.

Authors (Year)		Simulation conditions: Model/data, Sample sizes, and Parameters	Other conditions
Dong et al. (2018)^a^	M	*X*_1*jk*_, *Z*_1*k*_, *W*_1_, *W*_2_ dic. *V* (distr. varying per condition), *W*_2_^∗^*V*, *e_ijk_*, *u*_0*jk*_, *v*_00*k*_	Moderator slope variation: Non-randomly varying or random
	S	N_3_: 40, 80, N_2_: 5, 10, N_1_: 20	
	P	ICC_3_ = 0.15, ICC_2_ = 0.08, variance explained by *X*_1*jk*_, *Z*_1*k*_, *W*_1_: 50%, ES = 0.2 (*V*)	
McNeish and Wentzel (2017)	M	*X*_1*jk*_, *Z*_1*k*_, and *W*_1_∼*bern*(0.5), *X*_2*jk*_, *Z*_2*k*_, and *W*_2_∼*N*(0,1), *e_ijk_*, *u*_0*jk*_, *v*_00*k*_	Estimator: ML, REML or corrected REML; Model: three-level or misspecified as two-level model
	S	N_3_: 4, 7, 10, N_2_: 15 to 25, N_1_: 10 to 20	
	P	ICC_3_ = 0.05, 0.15, ICC_2_ = 0.2 ES = 0.1 (*X*_1*jk*_, *Z*_1*k*_, *W*_2_), ES = 0.2 (*X*_2*jk*_, *Z*_2*k*_, *W*_1_)	
		ε^2^*_e_* = 22, τ^2^_*u*0_ = 5.5, σ^2^_*v*0_ = 0.5 (ICC_3_ = 0.05) or σ^2^_*v*0_ = 2.5 (ICC_3_ = 0.15)	
Cunningham and Johnson (2016)^b^	M	*V* dic., *e_ijk_*, *u*_0*jk*_, *v*_00*k*_	–
	S	*N*_3_: 2 to 30, *N*_2_: 4, 6, 8, *N*_1_: 10, 20, 30	
	P	ICC_3_ = 0.03, ICC_2_ = 0.02	
de Jong et al. (2010)^c^	M	*X*_1*jk*_ (log-time), *Z_k_* dic. (model B only), *V* dic., *X*_1*jk*_^∗^*V*, *e_ijk_*, *u*_0*jk*_, *v*_00*k*_	Dropout: 0–25%, early, throughout the study duration, or late
	S	*N*_3_: 1–100, *N*_2_: 2, 4, 8, *N*_1_: 5, 11, 21	
	P	ICC_3_ = 0.18, ICC_2_ = 0.75, δ_000_ = 78.37/78.38 (model B), δ_100_ = -12.3/-10.56,	
		δ_010_ = -4.16, ε^2^*_e_* = 71.77/71.67, τ^2^_*u*0_ = 454.09/448.93, σ^2^_*v*0_ = 48.60/49.53, τ^2^_*u*1_ = 187.80/183.72	
Heo and Leon (2008)^b^	M	*W*_1_ dic., *e_ijk_*, *u*_0*jk*_, *v*_00*k*_	–
	S	*N*_3_: 4–40, *N*_2_: 5, 10, 25, 50, *N*_1_: 3, 6	
	P	ICC_3_ = 0.01, 0.05, 0.1, ICC_2_ = 0.4, 0.5, 0.6, δ_001_ = 0.3, 0.4, 0.5, δ_000_ = 0	
Konstantopoulos (2008)^a,c^	M	*W*_1_ dic., 5 covariates per level, *e_ijk_*, *u*_0*jk*_, *v*_00*k*_	Misspecification (two-level analysis)
	S	*N*_3_: 1–96, *N*_2_: 1–12, *N*_1_: 1–60	
	P	ICC_3_ = 0.1, 0.2, ICC_2_ = 0.067, 0.134, ES = 0.2, 0.5, variance explained by covariates: 50%	


*Outcome continuous if not otherwise stated. *N*_*1*_, level-1 sample size per level-2 unit; N_*2*_, level-2 sample size per level-3 unit; *N*_*3*_, level-3 sample size. ML, Maximum Likelihood; REML, Restricted Maximum Likelihood; ICC_*2*_, intraclass correlation coefficient of level-2; ICC_*3*_, intraclass correlation coefficient of level-3. ES, effect size. dic, dichotomous. *X_*ijk*_*, *Z_*jk*_*, *W_*1*_, e_*ijk*_*, *u_*0jk*_*, *u_*1jk*_*, *v_*00k*_, ε^*2*^_*e*_*, γ_00_, τ^2^_u0_, *τ^*2*^_*u1*_*, *σ^*2*^_*v0*_, δ_*000*_, δ_*100*_, δ_*010*_, δ_*001*_* refer to variables and effects on their respective level according to formulae (1) to (3). *V*, predictor with varying level according to condition. ^*a*^analytical solution only, ^*b*^ICC_*2*_ calculated as in (5), ^*c*^ICC_*2*_ calculated as in (6).*

**Table 3:** Selected results and recommendations of studies examining the impact of sample sizes on estimation results in two-level models.

Authors (Year)	Results	Sample size recommendations
Du and Wang (2016)	*N*_2_ ≥ 30: fixed effects *peb* < 0.05	*peb* < 0.05: *N*_2_ = 50 (ICC = 0.1) or 30 (ICC ≥ 0.2) for full data
	*N*_2_ ≤ 30 and ICC ≤ 0.2: τ^2^_*u*0_, ε^2^_e_ *peb* > 0.05	
	τ^2^_*u*0_ *peb* > ε^2^*_e_* *peb*	coverage: *N*_2_ = 50 for full data, otherwise *N*_2_ = 100
	*N*_2_ = 30 and 50% singletons: low coverage for fixed effects	
Spain et al. (2012)	**GLMM:** fixed effects estimates reliable, τ^2^_*u*0_ is over- (*N*_2_ > 100) or underestimated (*N*_2_ = 50), standard errors overestimated	*N*_2_ ≥ 100 for generally acceptable results, Laplace approximation is advised
	**HLM:** fixed effects estimates reliable for *N*_2_ > 50, τ^2^_*u*0_ slightly underestimated for *N*_2_ > 50	
Maas and Hox (2004)	**Normal distribution of level-2 errors:**	**Normal distribution of level-2 errors:**
	Fixed effects and r.e. variance component *peb* < 0.001 standard error coverage: 91–95%.	*N*_2_ ≥ 10 for fixed effects only
		*N*_2_ ≥ 30 for r.e. variance components
	**Non-normal distribution of level-2 errors:**	*N*_2_ ≥ 50 for standard errors
	Fixed effects and r.e. variance component *peb* negligible standard error coverage:	**Non-normal distribution of level-2 errors:**
		High number of groups
	fixed effect: 93–95%	Standard error estimates not interpretable
	ε^2^*_e_*: 94–96% (ML), 98–99% (MLR)	Non-parametric approach is advised
	τ^2^_*u*0_, τ^2^_*u*1_: 57–78% (ML), 80–92% (MLR)	
Maas and Hox (2005)	Fixed and random effects *peb* < 0.001	*N*_2_ ≥ 100 for τ^2^_*u*0_, τ^2^_*u*1_ standard errors
	15% underestimation of τ^2^_*u*0_, τ^2^_*u*1_ standard errors for *N*_2_ = 30.	
		*N*_2_ ≥ 10, *N*_1_ ≥ 5 for fixed effects
	**Non-coverage rate (5% nominal):**	
	fixed effect standard errors: 5–6%	
	ε^2^*_e_* standard errors: 5–6%	
	τ^2^_*u*0_, τ^2^_*u*1_ standard errors: 6–9%	
Moineddin et al. (2007)	Fixed effects overall *peb*: 0.01–0.04	*N*_2_ = *N*_1_ = 50 for valid effect estimates.
	τ^2^_*u*0_, τ^2^_*u*1_ overall *peb*: 0.05–0.07	*N*_2_ = 100, *N*_1_ = 50 do not suffice for robust τ^2^_*u*0_, τ^2^_*u*1_ standard errors
	Fixed effects and r.e. variance estimates *peb* ≥ 0.10 for *N*_2_ = 30/*N*_1_ = 5	
	**Standard error non-coverage rate (5% nominal):**	
	Fixed effect: 3–7%	
	τ^2^_*u*0_: 7–17%	
	τ^2^_*u*1_: 3–13%	
Mathieu et al. (2012)^a^	Average power across all conditions: 0.192	power to detect CLI can be increased by sampling more N_1_ units, rather than N_2_ units. N_1_ sample size is most important if additionally, lower level effects are of interest.
	CLI power mainly influenced by CLI effect size, N_1_, N_2_, and standard deviation of slopes.	
	power>80% for *N*_1_ = 18 and CLI effect size = 0.75 or *N*_1_ ≥ 18/*N*_2_ = 35	


**N*_*1*_, level-1 sample size per level-2 unit, *N*_*2*_, level-2 sample size. GLMM, generalized linear mixed model; HLM, hierarchical linear model; ICC, intraclass correlation coefficient; CLI, cross-level-interaction; *peb*, parameter estimation bias. ES, effect size. ML, maximum likelihood. MLR, maximum likelihood with robust standard errors. *X_*ij*_, Z_*j*_, e_*ij*_, u_*0j*_, u_*1j*_, ε^*2*^_*e*_, γ_*00*_, τ^*2*^_*u0*_, τ^*2*^_*u1*_* refer to variables and effects on their respective level according to formulae (1) and (2) with indices adjusted for two-level models. ^*a*^Only power for the CLI effect was investigated.*

**Table 4:** Selected results and recommendations of studies examining the impact of sample sizes on estimation results in three-level models.

Authors (Year)	Results	Sample size recommendations
Dong et al. (2018)^a^	Higher MDESD/smaller power for higher-level *V*	–
	Smaller MDESD/ larger power for *V* cont.	
	Power ≥80% for level-1/level-2 *V* dic. or any level *V* cont. and *N*_3_ = 80	
McNeish and Wentzel (2017)	Fixed effects: *peb* < 0.1; *peb* > 0.1 if level-3 is ignored	If the third level is incidental and not of research interest, a fixed-effects-only model is appropriate, as results are acceptable even for *N*_3_ = 4
	τ^2^_*u*0_ *peb* < 0.1, σ^2^_*v*0_ *peb* > 0.1	
	coverage < 0.9 for level-3 predictors	
	REML gives better results than ML	
Cunningham and Johnson (2016)^b^	*V* on level-1: power ≥ 0.9 for *N*_3_ > 2–12	–
	*V* on level-2: power ≥ 0.9 for *N*_3_ > 4–15	
	*V* on level-3: power ≥ 0.9 for *N*_3_ > 20–30	
de Jong et al. (2010)^c^	*V* on level-2, *N*_1_ = 11:	Sample sizes should be large at the level where randomization takes place (i.e., level-2 randomization requires larger N_2_, level-3 randomization requires larger N_3_)
	power ≥ 80% for *N*_3_ = 17/*N*_2_ = 8; *N*_3_ = 33/*N*_2_ = 4; *N*_3_ = 66/*N*_2_ = 2.	
	*V* on level-3, *N*_1_ = 11:	
	power ≥ 80% for *N*_3_ = 37/*N*_2_ = 8; *N*_3_ = 53/*N*_2_ = 4; *N*_3_ = 86/*N*_2_ = 2.	
	Numbers of measurements, adding a significant slope predictor or 25%	
	dropout have negligible effect on power.	
Heo and Leon (2008)^b^	ES = 0.3: power ≥ 0.8 for *N*_2_ = 5, *N*_3_ > 40; *N*_2_ = 25/50, *N*_3_ > 22	To increase power, more N_3_ should be sampled, since the impact of N_3_ on power is larger than N_2_ on power
	ES = 0.4: power ≥ 0.8 for *N*_2_ = 10, *N*_3_ > 17; *N*_2_ = 25/50, *N*_3_ > 13	
	ES = 0.5: power ≥ 0.8 for *N*_2_ = 5, *N*_3_ > 15; *N*_2_ = 10/25/50, *N*_3_ > 10	
	Empirical power < theoretical power	
Konstantopoulos (2008)^a,c^	Increasing N_3_ impacts power in all conditions	–
	N_1_ and N_2_ have non-substantial effect on power	
	Two-level analyses decrease power substantially	
	Including covariates increases power, e.g., *N*_3_ = 16/*N*_2_ = 2/*N*_1_ = 20,	
	ES = 0.5, ICC_3_ = 0.1, ICC_2_ = 0.067: power = 89% with covariates (power = 66% without covariates)	


**N*_*1*_, level-1 sample size per level-2 unit; *N*_*2*_, level-2 sample size per level-3 unit; *N*_*3*_, level-3 sample size. ML, Maximum Likelihood; REML, Restricted Maximum Likelihood; ICC_*2*_, intraclass correlation coefficient of level-2; ICC_*3*_, intraclass correlation coefficient of level-3. ES, effect size; MDESD, minimum detectable effect size difference; dic., dichotomous; cont., continuous. *τ^*2*^_*u0*_*, *σ^*2*^_*v0*_* refer to variables and effects on their respective level according to formulae (1) to (3). *V*, predictor with varying level according to condition. ^*a*^analytical solution only, ^*b*^ICC_*2*_ calculated as in (5), ^*c*^ICC_*2*_ calculated as in (6).*

### Sample Size and Estimation Quality of Fixed Effects Coefficients

Simulations for two-level models by Maas and Hox (2004, 2005; one normally distributed predictor per level with ES = 0.3, cross-level-interaction, random slope, and ICC ≤ 0.3) show that bias for medium sized fixed effects is usually below *peb* = 0.0005 for samples with at least five level-1 units each in 30 higher-level units. For the same conditions, Maas and Hox (2004) further show that coverage probabilities for the fixed effects remain within the acceptable range of 91–98%. For dyadic data, Du and Wang (2016) found negligible fixed effects bias for 30 or more dyads for different ICC levels (0.1–0.7) and a model with predictors on each level (ES = 0.3, fixed intercept ES = 1), including two-way and three-way interactions, and random intercepts (level-1 residual variance ES = 0.5). Coverage rates for fixed effects were generally acceptable for at least 50 dyads. Moreover Spain et al. (2012) investigated dyadic data with a binary outcome based on existing data from the health sector (five level-1 predictors with true values between -0.05 and 0.19, τ^2^_*u*0_ = 0.43, no level-2 predictors, no interactions, no random slopes), and found considerable bias for fixed effects estimates for less than 100 dyads, and severe under- or overestimation of standard errors.

For three-level models, bias of level-1 and level-2 predictors (continuous and discrete predictors with ES = 0.1 or ES = 0.2, ICC_3_ = 0.05 or 0.15, ICC_2_ = 0.2) has been found to fall below *peb* = 0.10, even if the level-3 sample size is smaller than 10 units (McNeish and Wentzel, 2017).

### Sample Size and Estimation Quality of Random Effects Coefficients

Previous simulation studies show that r.e. variance estimates are generally more severely biased than estimates for fixed effects. Most importantly, the severity of the bias depends on the sample sizes, the statistical model, and the level of the effect. For example, Maas and Hox (2005, see Table 1 for conditions) found a bias of *peb* < 0.001 for level-1 r.e. variance estimates (true value ε^2^_*e*_ = 0.5) in samples with 5 to 50 level-1 units each in 30 to 100 level-2 units, whereas Moineddin et al. (2007) used a two-level logistic regression model with one medium sized (ES = 0.3), normally distributed predictor at each level (ICC = 0.04 to 0.38), and found a bias of *peb* ≥ 1.00 for the level-2 random intercept variance estimate in small samples (30 to 50 groups with 5 units each). As a general rule, level-1 residual variance estimates are less biased than higher-level r.e. variance components, as shown by Maas and Hox (2004, see Table 1), who report sufficient coverage for level-1 residual variance estimates, but coverage below 80% for level-2 r.e. variance estimates in samples with at least 5 level-1 units each in 30 level-2 units. For analyses of dyadic data (Spain et al., 2012; Du and Wang, 2016; see Tables 1, 3), the inclusion of random effects is limited, since there need to be more units per cluster in the data than specified random variables in order for the model to be identified (Kenny et al., 2006; Du and Wang, 2016).

In three-level models, McNeish and Wentzel (2017) found the level-2 random intercept variance estimate (true value τ^2^_*u*0_ = 5.5) to be unbiased (*peb* < 0.10), even in samples with less than ten level-3 units, while the level-3 random intercept variance estimate (true value σ^2^_*v*0_ = 0.5 for ICC_3_ = 0.05, or σ^2^_*v*0_ = 2.5 for ICC_3_ = 0.15) in such samples is more heavily biased (see Table 2 for further conditions).

### Sample Size Allocation Across Levels

In addition to the overall sample size, the allocation of units across levels also influences the estimation results. Research on the sample size allocation has shown that generally, increasing the higher-level sample size leads to improved estimation accuracy in terms of both fixed effects and r.e. variance estimates on all levels (e.g., Maas and Hox, 2004, 2005, for two-level models; Konstantopoulos, 2008 for three-level models). If randomization is used to investigate a treatment effect, the total required sample size depends on the level of randomization. Generally, overall larger samples are required for higher-level randomization (Cunningham and Johnson, 2016; Hooper et al., 2016), and increasing the sample size at the level of randomization most strongly improves power (de Jong et al., 2010; longitudinal model with log-time at level-1, a level-2 covariate, ICC_3_ = 0.18, and ICC_2_ = 0.75, see Table 2 for further conditions). Heo and Leon (2008) derived formulae for power calculations to investigate necessary sample sizes to detect a level-1 effect in models with randomization at level three. They found that the required level-3 sample size is strongly (negatively) related to the size of the effect (ES = 0.3, 0.4, or 0.5), and showed that increasing the level-3 sample size is much more efficient to reliably (power ≥80%) detect the level-1 effect than increasing the level-2 sample size. Dong et al. (2018) further showed that power to detect a medium sized moderation effect in three-level models with a treatment-effect variable on level-3 (varying level of the moderator, ICC_3_ = 0.15, ICC_2_ = 0.08, including covariates on each level) generally depends on the level-3 sample size, although the level-1 sample size is also important for detecting lower level moderator effects. Aside from these findings, Snijders (2005) states, as a general rule, that the sample size of the level at which a particular variable is measured is most important for estimation accuracy of that variable’s effect. Regarding missing values, Hox (2013) further states that missing data at level one poses no problem for the analysis results, but missing higher-level data may require additional steps, such as multiple imputation, for reliable inference.

### Available Sample Size Recommendations

As multilevel analysis results are influenced by various factors, recommendations with regards to sample size strongly depend on the data, especially the sizes of the effects and model characteristics. Therefore, recommendations summarized in Table 3 (two-level models) and Table 4 (three-level models) relate to the simulation conditions in Tables 1, 2. In most simulation studies concerned with three-level models, findings were compared to existing recommendations for two-level models. Therefore, we briefly outline important results for two-level models, followed by results for three-level models. In summary, the most important findings are:

(i) In two-level models: Maas and Hox (2005) suggest a higher-level sample size of at least 100 for accurate estimation of higher-level r.e. variance components and their standard errors (see Table 1 for conditions). However, they argue that smaller level-2 sample sizes might be sufficient in practice, as they showed that sampling 50 level-2 units provides acceptable results for higher-level r.e. variance estimates, with a non-coverage rate of 7.3% as opposed to the nominal 5%. Mathieu et al. (2012) investigated cross-level-interactions in models with one normally distributed predictor per level (ICC: 0.15 or 0.30; various ES ≤ 0.75), and recommend sampling more thoroughly on level-1, rather than on level-2, especially if lower level effects are of interest in addition to the cross-level-effect. For dyadic data, Du and Wang (2016) recommend at least 50 dyads in order to reliably estimate fixed effects and r.e. variance components in case of non-missing data (see Table 1 for conditions). In case of missing data (10 to 50% of singletons), they recommend at least 100 dyads. They point out, however, that such sample sizes might not be sufficient for adequate power to detect effects. Recently, Lane and Hennes (2018) developed and demonstrated a guide to determine power for a user specified dyadic data model.
(ii) In three-level models: de Jong et al. (2010) showed that sufficient power for large fixed effects can be reached with relatively small level-2 sample sizes given that the level-3 sample size is large, such as 17 level-3 units with 8 level-2 units each, or 66 level-3 units with 2 level-2 units each (see Table 2 for conditions). However, they argue that estimates for both coefficients and their standard errors could be strongly biased for these small level-2 sample sizes. McNeish and Wentzel (2017) show that if the third level is not of research interest, but solely included due to the sampling procedure, four level-3 units can ensure sound estimation results and sufficient coverage rates at level-2 and level-1 (see Table 2 for conditions). To detect a level-1 effect of a dichotomous variable in a three-level model with randomization at level three (ICC_3_ = 0.01, 0.1, ICC_2_ = 0.4, 0.5, 0.6), Heo and Leon (2008) found that effects of size ES = 0.3 with fewer than 10 level-1 and level-2 units require more than 30 level-3 units for sufficient power. For larger level-2 sample sizes (*N*_2_ = 25), 6 level-3 units can be sufficient. To find larger effects of size 0.5, overall small sample sizes can be sufficient (e.g., *N*_1_ = 6, *N*_2_ = 5, *N*_3_ > 9). Cunningham and Johnson (2016) furthermore found that for an effect of ES = 1.8, a level-2 sample size of 4 to 8, and a level-1 sample size of 10 to 30, a power of 90% can be reached with less than 12 level-3 units when randomization takes place at level-1, and a few more required units in case of level-2 randomization, while between 20 and 30 units are required if randomization takes place at the highest level.

### Aim of This Study and Research Questions

In summary, previous studies have focused mainly on estimation quality in two-level models, and recent findings on three-level models reveal a complex interplay of factors influencing estimation quality. Hence, the soundness of fixed effects and r.e. variance estimates in three-level models is not sufficiently well known. In particular, it is not clear if there are differences in the estimation quality between level-2 and level-3 model parameters depending on the overall sample size and allocation of observational units to the different levels. Yet, this information is of high importance, since sample size recommendations for three-level models with repeated measures are sparse, with even fewer studies including a longitudinal trend (e.g., linear or quadratic trajectories) and higher order clustering. Moreover, most simulation studies have generated data following theoretical considerations rather than empirical findings. As a consequence, researchers who analyze empirical three-level data lack information on how large their sample sizes need to be for each data level, and how sample size recommendations from artificially created datasets apply to their research setting.

The present simulation study helps researchers in planning their study with respect to the sampling procedure by comparing estimates obtained under various sample size conditions to known true values of population data. We use data from a typical research setting in developmental psychology, yielding practice-relevant samples, effects, and models. We examine how sample sizes and missing values affect estimation quality of the MLA for fixed effects (including cross-level-interactions) and r.e. variance estimates across the three data levels. By using data reflecting typical MLA-results and sample characteristics of an illustrative psychological study, we aim to help researchers in similar research settings decide on the appropriate sample sizes. As a consequence, results and their derived recommendations are intended for models and data which are comparable to the conditions in these analyses.

## Materials and Methods

### The Illustrative Study

The study published by Maulana et al. (2013) serves as a suitable basis for our simulation study for several reasons:

(i) the research setting – observing children clustered within classes over a period of time – is common in educational research
(ii) the study provides sufficient methodological information in order to recreate comparable data
(iii) the multilevel model of the study uses three-level-data including predictors on each level, a continuous dependent variable, random slopes, and intercepts on levels two and three, and repeated measures at the first level
(iv) the study depicts a realistic sampling process with typical samples and analysis results, which are transferable to similar research contexts

In the study, the authors investigated the link between student-teacher interpersonal relationship, academic motivation, and its development over time by surveying 504 Indonesian first-grade secondary students (level 2) from 16 classes (level 3) about their academic motivation and perceived influence and proximity of their teachers over the course of one school-year with five measurement occasions (level 1). Our analyses are based on Model 2 in Maulana et al. (2013, p. 472), which assesses how motivation develops over time, and how it is affected by perceived teacher influence at each measurement occasion (level-1), gender (level-2), subject taught (level-3), and classtype (level-3). For our population data and model, we removed the predictor *subject taught* in order to obtain a realistic, yet concise model depicting a growth process with one covariate at every level. Table 5 depicts the analysis results as reported in Table 4 of Maulana et al. (2013) and indicates which variables are used for the population data and analyses. In line with the original study, we included random intercepts and random slopes for the linear effect of time at both higher levels. Equations (9) to (11) depict the resulting three-level model:

Level 1:

(9) $${Motivation}_{ijk}=\beta_{0jk}+\beta_{1jk}{Time}_{ijk}+\beta_{2jk}{Time}_{ijk}^{2}+\beta_{3jk}{Influence}_{ijk}+e_{ijk}$$

Level 2:

(10) $$\begin{array}{c} \beta_{0jk}=\gamma_{00k}+\gamma_{01k}{Gender}_{jk}+u_{0jk}\\ \beta_{1jk}=\gamma_{10k}+\gamma_{11k}{Gender}_{jk}+u_{1jk}\\ \beta_{2jk}=\gamma_{20k}\\ \beta_{3jk}=\gamma_{30k} \end{array}$$

Level 3:

(11) $$\begin{array}{c} \gamma_{00k}=\delta_{000}+\delta_{001}{Classtype}_{k}+v_{00k}\\ \gamma_{01k}=\delta_{010}\\ \gamma_{10k}=\delta_{100}+\delta_{101}{Classtype}_{k}+v_{10k}\\ \gamma_{11k}=\delta_{110}\\ \gamma_{20k}=\delta_{200}\\ \gamma_{30k}=\delta_{300}+\delta_{301}{Classtype}_{k} \end{array}\,\left(11\right)$$

**Table 5:** ML growth curve results by Maulana et al. (2013).

	Influence on controlled motivation	
	
Variable	Coefficient	*SE*
Fixed effects level 1	
**intercept**	**3.2032^∗∗∗^**	0.0860
**time**	**0.0547^∗∗^**	0.0205
**influence**	**0.1493^∗^**	0.0694
Fixed effects level 2	
**gender**	**0.0072**	0.0647
Fixed effects level 3	
subject taught	0.0314	0.0711
**classtype**	**0.2946^∗∗∗^**	0.0781
Interaction effects	
**time × classtype**	**0.0092**	0.0107
time × subject taught	-0.0055	0.0106
**time × gender**	**0.0056**	0.0081
**time × time**	-**0.0039^∗∗^**	0.0015
**influence × classtype**	-**0.1514**	0.0848
Random variance level 3	
**intercept**	**0.0041**	0.0073
intercept × time	0.0001	0.0008
**time**	**0.0002**	0.0002
Random variance level 2	
**intercept**	**0.1950**	0.0346
intercept × time	-0.0069	0.0040
**time**	**0.0009**	0.0006
Random variance level 1	
**residual**	**0.2622**	0.0123


**SE*, standard error. Bold components indicate variables and effects included in the data generating process. Sample size = 1892. ^∗^*p* < 0.05, ^∗∗^*p* < 0.01, ^∗∗∗^*p* < 0.001.*

### The Experimental Design

In order to investigate how the parameter estimates in MLA are affected by the sampling plan and missing data, we vary three factors: The level-3 sample size (number of classrooms: *N*_3_ = 15, 35, 55), the level-2 sample size (number of students per class: *N*_2_ = 5, 15, 35), and the level-1 sample size by means of missing value patterns: (i) no missing values (complete data: COM), (ii) 20% of students missing level-1 reports completely at random at arbitrary measurement occasions (this pattern is referred to as: MCAR), and (iii) 20% of students dropping out and thus not reporting level-1 variable values for the last two measurement occasions (DrOP2), or (iv) the last three measurement occasions (DrOP3). The 20% of missing data in the drop-out conditions DrOP2 and DrOP3 are also missing completely at random. To assess how our sample size conditions compare to sample sizes of recent studies in the educational setting, we conducted a non-exhaustive literature search of relevant studies. We limited our search to include studies which (1) were published in academic journals in 2013 or more recently, (2) are listed in PsychINFO, (3) use either three-level multilevel analysis or multilevel growth curves, and (4) analyze data in the school context. We identified 20 studies which sampled and analyzed data according to our criteria and found median sample sizes of 49 schools (min = 10, max = 933), 68 classes (min = 16, max = 565), 1943 children or adolescents (min = 55, max = 29153), and four measurement occasions (min = 2, max = 14). We further identified 30 studies analyzing pre-existing, large-scale data (e.g., PISA, TIMSS) and found median sample sizes of 259 schools (min = 44, max = 11075), 594.5 classes (min = 103, max = 18761), 7718 children or adolescents (min = 1698, max = 470000), and four measurement occasions (min = 3, max = 5). Hence, our sample size conditions, ranging from 75 students in 15 classes to 1925 students in 55 schools, lie within the range of recently analyzed sample sizes in the field. The percentage of missing values was chosen based on typically reported attrition rates in research concerning the educational system (e.g., Wagner et al., 1997; Miles and Stipek, 2006; Zvoch and Stevens, 2006; Konstantopoulos and Chung, 2011). Table 6 describes the missing value patterns by depicting the percentage of data used for analysis at each measurement occasion. In total, the simulation study encompasses 3 × 3 × 4 = 36 conditions.

**Table 6:** Percentage of students data used for analysis for each missing value pattern.

	T0	T1	T2	T3	T4
COM	100%	100%	100%	100%	100%
MCAR	R	R	R	R	R
DrOP2	100%	100%	100%	80%	80%
DrOP3	100%	100%	80%	80%	80%


*T0, baseline measurement, T1 to T4 denote successive measurements. COM to DrOP3 correspond to missing value patterns described in the section “Materials and Methods.” *R* = 20% of overall data is missing completely at random.*

### The Data Generating Process

Firstly, we generated the population data using version 14.1. of Stata (StataCorp, 2015) according to the information provided in the illustrative study. In total, we generated the population data to consist of 3000 classrooms, with 1000 classrooms for each of the three N_2_ (5, 15, or 35) conditions. We used the variable means, standard deviations, and distributions reported in the illustrative study to generate values for the independent variables, and used the reported MLA results (i.e., fixed effects estimates and r.e. variance estimates) to generate values for the dependent variable (see Equations 9 to 11). The independent variables are gender (56% girls; binary), classtype (52% heterogeneous-ability classes; binary), time passed since baseline measure in months (0, 1.5, 4, 7, and 10), and interaction terms. Since the illustrative study did not report non-normal data, the metric variable influence was modeled as normally distributed with *M* = 0.42 and *SD* = 0.36. Random effects are also modeled as normally distributed variables with mean zero and variances equal to those reported in the illustrative study.

In a next step, we realized samples from the population data according to the N_2_ (number of students per class) and N_3_ (number of classrooms) conditions with full data (missing value pattern: COM). For each N_3_/N_2_ combination, we drew the desired number of classes (i.e., 15, 35, or 55) from the 1000 classes in the population data that contained the desired number of students (i.e., 5, 15, or 35). The classes for each realized sample were drawn without replacement, such that no particular simulated sample contains duplicates of level-3 units. We repeatedly drew samples to obtain 1000 datasets for each of the 3 × 3 × 1 = 9 conditions (N_2_ × N_3_ conditions) with complete data. For the missing value patterns MCAR, DrOP2, and DrOP3, we repeated the data generation process described above separately for each of the three patterns, and in an additional step deleted values according to the desired condition (see section “The Experimental Design”). As a result, we obtained 1000 samples for each of the 3 × 3 × 3 = 27 conditions with missing values.

### Analysis Procedure

All analyses were carried out using R (R Development Core Team, 2016). In a first step, the true parameters from the population were obtained by applying the lmer()-function of the LME4-package (Bates et al., 2016). To assess variability due to clustering in the population data, ICC values for levels two and three were computed as in (4) to (6). Notably, since the population r.e. variances are modeled according to the values reported in the illustrative study, the ICC values are evaluated but not varied as an additional analysis condition in this study. In order to more easily interpret the original study and evaluate its results, effect size values for fixed effects and r.e. variances are advantageous. However, the reduction of variability on each level as a measure of effect size is not applicable for longitudinal data (Hox et al., 2017), and available effect sizes in multilevel models have been developed for measures of mean differences between groups (e.g., Tymms, 2004; Hedges, 2011) only. In order to nonetheless provide a general understanding for the magnitude of effects in our analyses, we used the approach by Tymms (2004) to calculate effect sizes as the regression weight relative to the remaining residual variance of the full model. Hence, the resulting values are not exact effect sizes, and should therefore only serve as an approximate indicator of the magnitude of effects within this study. (12) and (13) provide formulae for dichotomous and continuous variables, respectively, for a given regression coefficient β, the predictor variable standard deviation *SD_Predictor_*, and the level-1 residual variance ε^2^_*e*_.

(12) $${ES}_{dichotomous}=\frac{\beta }{\varepsilon_{e}^{2}}$$

(13) $${ES}_{continuous}=\frac{2^{*}\beta^{*}{SD}_{\mathrm{Pr}edictor}}{\varepsilon_{e}^{2}}$$

For the r.e. variances, Tymms (2004) recommends using their close relationship to the ICC and calculate the level-wise effect size for the random part of the model as in (14). This formula is applicable for ICC values (4) to (6) presented above.

(14) $${ES}_{random}=\sqrt{\frac{4^{*}ICC}{1-ICC}}$$

After evaluating the population data, the samples were analyzed using the same model and lmer()-function. For each condition, the *peb* over all 1000 simulated samples was then calculated for all fixed effects and r.e. variance estimates. Since the LME4-package does not provide *p*-values for multilevel analysis results^1^, the 95%-confidence intervals were constructed (see Wald-method in Bates et al., 2016) for fixed effects. For each parameter of fixed effects individually, coverage was evaluated by whether it ranges between 91% and 98%, and power was assessed for each predictor in each condition by the percentage of simulated samples with the confidence interval not including the value zero.

## Results

### Population Model

The data generation process yielded a population that corresponds very closely to the sample reported by Maulana et al. (2013) with respect to descriptive statistics (see Table 7) and model parameters of the MLA using the full population data (see Table 8). Perceived influence scores across measurements are equal to the values reported in the illustrative study (*M* = 0.42, *SD* = 0.36), and motivation scores differ only by 0.01 points in mean (*M* = 3.54) and 0.03 points in standard deviation (*SD* = 0.73). 55.67% of the population consists of girls (56% in the illustrative study), and 50.70% of the classrooms are heterogeneous (52% in the illustrative study).

**Table 7:** Population characteristics and values reported by Maulana et al. (2013).

	*M* [Study *M*]	*SD* [Study *SD*]	Fraction [Study fraction] in %
Influence^a^	0.42 [0.42]	0.36 [0.36]	–
Motivation^a^	3.54 [3.55]	0.73 [0.70]	–
Gender^b^			
girls	–	–	55.67 [56]
boys	–	–	44.33 [44]
Classtype^c^			
heterogeneous	–	–	50.70 [52]
homogeneous	–	–	49.30 [48]


**M*, mean; *SD*, standard deviation. Values reported by Maulana et al. (2013) indicated in brackets. ^*a*^Based on 275000 reports. ^*b*^Based on 55000 students. ^*c*^Based on 3000 classrooms.*

**Table 8:** Results of the MLA for the population.

	Model: influence on controlled motivation^a^
	
Variable	Coefficient
**Fixed effects level 1**	
intercept	3.2110
time	0.0529
influence	0.1465
**Fixed effects level 2**	
gender	0.0083
**Fixed effects level 3**	
classtype	0.2845
**Interaction effects**	
classtype × time	0.0100
gender × time	0.0057
influence × classtype	-0.1419
time × time	-0.0038
**Random variances level 3**	
intercept	0.0045
time	0.0002
**Random variances level 2**	
intercept	0.1926
time	0.0009
**Random variance level 1**	
residual	0.2626


*^*a*^Population size = 275000.*

Table 8 presents the results of the MLA analysis of the whole population, which serve as true values for subsequent analyses. The largest deviations from the original study are 0.0101 points for fixed effect estimates (classtype: population coefficient = 0.2845) and 0.0004 points for r.e. variance estimates (level-3 intercept: population variance = 0.0045).

Table 9 presents the r.e. variance estimates of the population empty model and the resulting ICC values. As can be seen, stochastic dependency in the data due to clustering mainly occurs with regards to the level-2 intercepts (using equation (15): ICC = 0.44; using equation (16): ICC = 0.39).

**Table 9:** Random effects variance components and ICC values for the multilevel model without predictors (empty model) for the population data.

	Variance component	ICC
Level-1 (Residual)	ε^2^*_e_* = 0.30	–
Level-2 Intercept	τ^2^_*u*0_ = _._20	ρ_level2 (a)_ = 0.44 ρ_level2 (b)_ = 0.39
Level-3 Intercept	σ^2^_*v*0_ = 0.03	ρ_classes_ = 0.05


*ICC, intraclass correlation coefficient. Version (a) of the level-2 Intercept ICC is calculated according to (5), (b) is calculated according to (6).*

Table 10 presents the estimated effect sizes for each predictor following Tymms (2004). In a simulation study, Tymms (2004) showed that large effect sizes, such as for time, time × time, and classtype, are slightly underestimated (below 10% of underestimation). The results in Table 10 show a range of small and large effects for both main effects (e.g., ES*_gender_* = 0.02; ES_time_ = 0.75) and interaction effects (e.g., ES_gender × time_ = 0.08; ES_time × time_ = -0.55). As the coefficients of this case study are subject of evaluation and the effect sizes cannot be calculated accurately, further analysis results will refer to regression coefficients rather than effect sizes.

**Table 10:** Effect sizes of predictors and random effects variance components.

	Regression coefficient	standard deviation of predictor variable	Effect size^a^
Predictors level 1	
time	0.05	3.63	0.75
influence	0.15	0.36	0.21
Predictor level 2	
gender	0.01	–	0.02
Predictor level 3	
classtype	0.28	–	0.56
Interaction effects	
time × time	-0.0038	37.51	-0.55
gender × time	0.01	3.51	0.08
classtype × time	0.01	3.40	0.13
influence × classtype	-0.14	0.33	-0.18

**Random effects**	**Variance component^b^**	**ICC**	**Effect size^b^**

Level-2 intercept (a)	0.20	0.44	1.76
Level-2 intercept (b)	0.20	0.39	1.59
Level-3 intercept	0.03	0.05	0.46


*^*a*^Based on Tymms (2004) with ε^*2*^_*e*_ from the full model. ^*b*^Variance components and, hence, effect sizes are based on the empty model. ICC, intraclass correlation coefficient. Random slopes are excluded as they are not part of the empty model used to calculate ICC values.*

### Estimation Bias: Fixed Effects

In samples without missing values (COM; see Table 11), the *peb* consistently falls below 0.10 for the following regression coefficients^2^: intercept, time, and time × time. For conditions with *N*_2_ = 5, coefficients are underestimated for the level-3 effect classtype (*peb* ranging from-0.16 to -0.13), level-1 effect influence (*N*_3_ = 35: *peb* = -0.10, *N*_3_ = 55: *peb* = -0.12), the cross-level-interactions classtype × time (*peb* between -0.25 and -0.20), and classtype × influence (*N*_3_ = 55: *peb* = -0.14). Coefficients for gender (level-2) are consistently overestimated in *N*_2_ = 5 (*peb* between 0.35, and 0.99) and *N*_2_ = 15 (*peb* between 1.13, and 1.47), but underestimated for *N*_2_ = 35 (*peb* ranging from -0.76 to -0.26). The cross-level interaction gender × time, in contrast, is underestimated for *N*_2_ = 5 (*peb* between -0.55 and -0.41) and *N*_2_ = 15 (15 classes: *peb* = -0.14; 55 classes: *peb* = -0.15), whereas it is overestimated for *N*_2_ = 35/*N*_3_ = 35 (*peb* = 0.14) and *N*_2_ = 35/*N*_3_ = 55 (*peb* = 0.11). For fixed effects, *peb* is mainly improved by increasing the number of students per classroom, with gender (level-2) requiring at least *N*_2_ = 35 to substantially improve estimation quality.

**Table 11:** Parameter estimation bias (peb) of samples without missing values for fixed effects.

	Level 1			Level 2	Level 3	Interaction effects			
				
N_2_/N_3_	Intercept *peb*	Time *peb*	Influence *peb*	Gender *peb*	Classtype *peb*	time × time *peb*	classtype × time *peb*	gender × time *peb*	influence × classtype *peb*
5/15	0.003	0.056	-0.054	**0.987**	**-0.129**	0.011	**-0.248**	**-0.554**	0.003
5/35	0.005	0.003	**-0.100**	**1.671**	**-0.155**	-0.040	**-0.197**	**-0.551**	-0.087
5/55	0.006	0.025	**-0.117**	**0.347**	**-0.162**	-0.014	**-0.234**	**-0.405**	**-0.138**
15/15	-0.003	0.007	0.024	**1.474**	0.026	0.002	0.082	**-0.138**	0.017
15/35	-0.002	0.007	0.032	**1.178**	0.035	0.011	**0.104**	-0.088	0.063
15/55	-0.003	0.019	0.028	**1.127**	0.038	0.025	0.094	**-0.151**	0.049
35/15	< 0.001	-0.012	0.002	-**0.264**	0.012	-0.009	0.034	0.056	-0.012
35/35	0.001	-0.011	0.010	**-0.760**	0.012	< 0.001	0.031	**0.137**	0.004
35/55	< 0.001	-0.006	0.011	**-0.702**	0.015	0.001	0.045	**0.110**	0.004


**N*_*2*_, Number of students per class (level-2 sample size). *N*_*3*_, Number of classes (level-3 sample size). Bolded numbers indicate *peb* > 0.1.*

In samples with missing values or dropout (MCAR, DrOP2, DrOP3), *peb* does not differ substantially compared to samples without missing values (COM; see Supplementary Tables A1–3). For gender, the *peb* decreases slightly for most sample sizes, but does not fall above *peb* = -0.25 (*N*_2_ = 35/*N*_3_ = 15 in MCAR), staying close to the results for samples without missing values (*peb* = -0.26, in the same sample size). *peb* further decreases for most interaction effects in samples with *N*_2_ = 5 and MCAR, with the largest decrease for gender × time in *N*_2_ = 5/*N*_3_ = 35 (no missing values: *peb* = -0.55; missing values: *peb* = -0.43).

### Estimation Bias: Random Effects

Table 12 provides *peb* values for r.e. variance estimates in samples without missing values (COM; for results with missing values, see Supplementary Tables A4–6). The bias exceeds *peb* = 0.10 for the r.e. variance estimate of the level-3 intercept in all conditions but *N*_2_ = 35/*N*_3_ = 35 (range: *peb* = -0.11 in *N*_2_ = 35/*N*_3_ = 55 to *peb* = 1.94 in *N*_2_ = 5/*N*_3_ = 15). The r.e. variance estimate of the level-3 slope is overestimated for *N*_2_ = 5/*N*_3_ = 35 (*peb* = 0.46), *N*_2_ = 5/*N*_3_ = 55 (*peb* = 0.20), and *N*_2_ = 15/*N*_3_ = 15 (*peb* = 0.17), whereas the r.e. variance estimate of the level-2 slope is only overestimated in *N*_2_ = 5/*N*_3_ = 15 (*peb* = 0.10). For all other sample sizes and variables, bias is below 0.10. For r.e. variance estimates, *peb* is mostly reduced by increasing the number of students per class.

**Table 12:** Parameter estimation bias (peb) of samples without missing values for variances of random effects.

N_2_/N_3_	Level-3 intercept *peb*	Level-3 slope *peb*	Level-2 intercept *peb*	Level-2 slope *peb*	Level-1 residual *peb*
5/15	**1.944**	**0.455**	-0.067	**0.104**	0.002
5/35	**0.766**	**0.200**	-0.066	0.060	0.007
5/55	**0.389**	0.063	-0.060	0.090	0.011
15/15	**0.795**	**0.173**	-0.025	-0.040	0.001
15/35	**0.419**	0.099	-0.014	-0.031	< 0.001
15/55	**0.307**	0.084	-0.014	-0.034	0.002
35/15	**0.108**	0.010	0.004	-0.014	-0.001
35/35	-0.078	-0.028	0.012	-0.009	-0.002
55/55	-0.105	-0.028	0.013	-0.013	-0.002


**N*_*2*_, Number of students per class (level-2 sample size per group). *N*_*3*_, Number of classes (level-3 sample size). Bolded numbers indicate *peb* > 0.1.*

For samples including missing values, most *pebs* do not change by more than 0.01, except for the level-2 random slope variance in samples with *N*_2_ = 5 and MCAR (*N*_3_ = 15: *peb* = 0.20; *N*_3_ = 35: *peb* = 0.09; *N*_3_ = 55: *peb* = 0.02) showing an increase of up to 0.09. Results for the level-3 r.e. variances are mixed. For the r.e. variance estimate of the level-3 intercept, mean bias decreases for *N*_2_ = 35/*N*_3_ = 55 and MCAR (*peb* = -0.07) with *peb* below 0.10. In most other conditions, *peb* of the r.e. variance estimate of the level-3 intercept becomes larger, with increases of more than 1.00 in *peb* for MCAR. Increases in *peb* for the r.e. variance estimate of the level-3 slope mainly concern sample sizes with *N*_2_ = 5 and additionally *N*_2_ = 15 in samples with MCAR. For *N*_2_ = 5/*N*_3_ = 15, *peb* reaches 0.69. In total, missing values in *N*_2_ = 35 do not lead to considerable changes in the r.e. variance estimates, while missing values in *N*_2_ = 15 mainly affect level-3 effects. Missing values in *N*_2_ = 5 lead to stronger estimation bias in all conditions except for the level-1 residual variance estimates.

### Estimation Fluctuations

**FIGURE 1:**
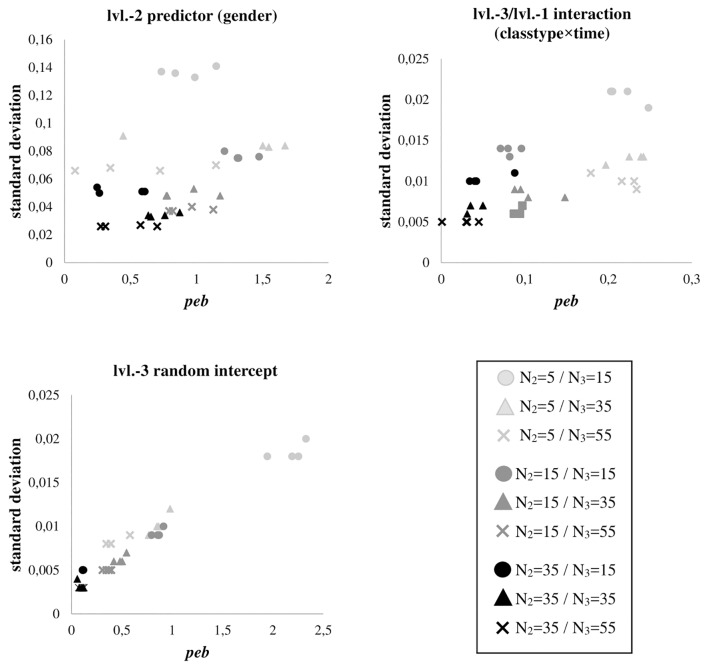
Parameter estimation bias (*peb*) for level-2 predictor gender, cross-level interaction classtype × time, and level-3 random intercept variance, in relation to the standard deviation of their estimated parameter values over 1000 simulation runs for each sample size condition. Shades differentiate between numbers of students per class (N_2_), shapes differentiate between numbers of classrooms per sample (N_3_). As results are very similar between samples with and without missing values, plots do not differentiate between missing value patterns.

The association of estimation fluctuations, measured by the parameters’ standard deviation over all replications, and *peb* expresses if stronger biases are related to less stable estimates. Figure 1 depicts these associations for gender, classtype × time, and the level-3 random intercept variance across all conditions (for all other effects, which show consistently comparable findings, see Supplementary Figures A1–3). Increasing the number of students per classroom generally leads to substantial improvements in estimation quality in terms of less estimation fluctuations (more efficiency) and *peb* (less bias). Moreover, for the smallest sample size at level-2, we find the largest *peb* and the largest standard deviations across the replications.

### Coverage

Coverage rates for all fixed effects fall within the acceptable range of 91% to 98%, with the lowest being *cov* = 91.5% for the level-3 variable classtype in *N*_2_ = 5/*N*_3_ = 55 with MCAR, and the highest being *cov* = 97.3% for the level-1 variable time in *N*_2_ = 35/*N*_3_ = 55 with DrOP2. Complete tabulated coverage values can be found in the Supplementary Tables A7–10.

### Statistical Power

**FIGURE 2:**
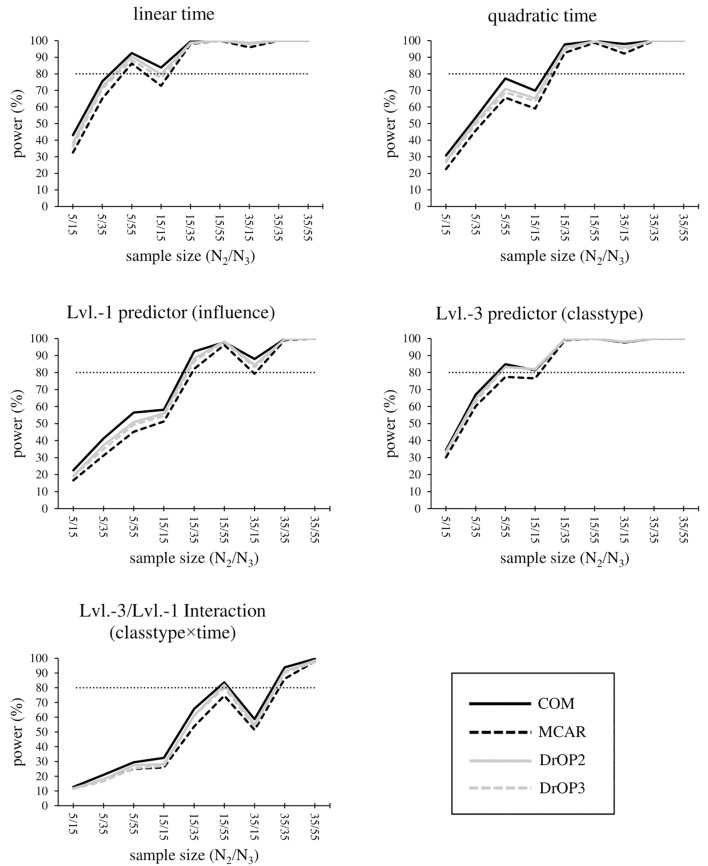
Statistical power for the time effect, level-1 predictor influence, level-3 predictor classtype, and the cross-level interaction of influence × classtype. The sample size condition is displayed as “level-2 sample size/level-3 sample size,” e.g., 5/15 corresponds to “5 students each in 15 sampled classrooms.” The missing value conditions (see section “Materials and Methods” and Table 6) are displayed by the type and shade of lines. The light dotted line marks the minimum sufficient power of 80%.

Overall, power for fixed effects is greater than 80% in all conditions for the level-1 regression intercept, but it is below 80% in any condition for the smallest effects: gender, gender × time and classtype × time. Power results for the remaining effects are summarized below and displayed in Figure 2. Full tabulated results can be found in the Supplementary Tables A11–12.

In complete samples (COM) with *N*_2_ = 35, effects are most reliably detected, with power ≥80% for all remaining effects except influence × classtype in *N*_2_ = 35/*N*_3_ = 15 (power = 58.7%). Sampling either *N*_2_ = 35/*N*_3_ = 35 or *N*_2_ = 35/*N*_3_ = 55 results in power >90% for all remaining effects. Additionally, sampling *N*_2_ = 15/*N*_3_ = 35 or *N*_2_ = 15/*N*_3_ = 55 provides sufficient power for the linear and quadratic time effect, classtype, and influence, whereas the cross-level interaction influence × classtype reaches power >80% for *N*_2_ = 15/*N*_3_ = 55 only. In order to statistically detect the effect of classtype, influence, and the quadratic time effect, *N*_2_ = 15/*N*_3_ = 35 is required, and time requires at least *N*_2_ = 5/*N*_3_ = 55, or alternatively *N*_2_ = 15 regardless of the N_3_ condition.

In conditions with missing values, power generally decreases with the most pronounced decrease for the MCAR-condition. DrOP2 results in the smallest decrease, where only one additional effect (the linear time effect in *N*_2_ = 15/*N*_3_ = 15) has a power below 80% (complete samples: power = 83.8%, DrOP2: power = 79.8%). As in samples without missing values, conditions *N*_2_ = 15/*N*_3_ = 35, 15/55, and samples with *N*_2_ = 35, provide sufficient power for the effects time, time × time, and classtype. This also holds true for the effect of influence, with the exception of *N*_2_ = 35/*N*_3_ = 15, where power = 79.3%. For classtype, however, power drops below 80% for MCAR in *N*_2_ = 5/*N*_2_ = 55 (power = 77.4%) and *N*_2_ = 15/*N*_3_ = 15 (power = 76.6%). For the interaction influence × classtype, power is still sufficient for *N*_2_ = 35/*N*_3_ = 35 and *N*_2_ = 35/*N*_3_ = 55, but drops below 80% for MCAR (power = 74.6%) and DrOP3 (power = 79.9%).

## Summary and Discussion

This simulation study sheds light on the quality of parameter estimates in MLA for data situations that are comparable to the one encountered by Maulana et al. (2013). The data was hierarchically structured on three-levels, with random effects on levels two and three, five measurement occasions, four main effects (Level-1 β_time_ = 0.05, ES = 0.75; Level-1 β_influence_ = 0.15, ES = 0.21; Level-2 β_gender_ = 0.01, ES = 0.02; Level-3 β_classtype_ = 0.28, ES = 0.56), four interaction effects (β_time × time_ < 0.01, ES = -0.55; β_classtype × time_ = 0.01, ES = 0.13; β_gender × time_ = 0.01, ES = 0.08; β_influence × classtype_ = -0.14, ES = -0.18), and a continuous response variable. We realized conditions differing in overall sample size, unit allocation, and missing value patterns, and investigated differences in estimation quality using MLA. When comparing analysis results across conditions, it has become apparent that in this setting, the quality of the analysis results strongly depends on the sample size, particularly the unit allocation to level-2, which describes the number of students. In the following subsections, we summarize the required sample sizes for sound estimation results, derive sampling recommendations, and discuss limitations and future prospects. In this regard, the recommendations presented are based on the specific simulation conditions in this study.

### Required Sample Sizes

The simulation study showed that all model parameters can be reliably estimated except for the small effect of gender, which requires sample sizes greater than those examined. This is most plausibly due to its standard error being equally large as the model parameter. Results for interaction effects are mixed, with some cross-level-interactions requiring at least 15 level-2 units (gender × time, classtype × time), and both time × time and influence × classtype being unbiased even in small samples with 5 level-2 and 15 level-3 units. The results for the r.e. variance estimates are mostly consistent with previous studies (e.g., Maas and Hox, 2004, 2005; Moineddin et al., 2007; McNeish and Wentzel, 2017). Bias is very high for level-3 r.e. variance estimates, even for large sample sizes, but smaller for level-2 r.e. variance estimates. Bias of level-1 residual variances is negligible with *peb*s < 0.015. Heavy fluctuations in the level-3 r.e. variance estimates might also be due to their small size.

The results show that there is a minimum number of classrooms (level-3 sample size) needed for stable results, as results for samples with only 15 classrooms are most strongly biased due to strong fluctuations in estimates. Notably, studies on different three-level models report far smaller level-3 sample sizes to be sufficient in terms of *peb* (Heo and Leon, 2008; Cunningham and Johnson, 2016; McNeish and Wentzel, 2017). Apart from that, the number of students per class (level-2 sample size) is consistently more important than adding more classrooms to the sample, with especially low estimation quality in samples with only five students per class. Specifically, the highest-level sample size in this study does not impact results most, as the majority of previous studies suggests (e.g., Maas and Hox, 2004, 2005; Konstantopoulos, 2008; Heo and Leon, 2008; Scherbaum and Ferreter, 2009; Dong et al., 2018). This conflicting result might be due to a less pronounced multilevel-structure, as the r.e. variance components on classroom-level and, consequently, the ICC, are very small. Furthermore, for the level-3 predictor, the number of classrooms seems to be as important as the number of students. This is at least in parts in line with previous findings stating that a parameter is estimated most accurately if the sample size at the corresponding level is sufficiently large (Snijders, 2005). In addition to the *peb* as a measure of bias, we evaluated the strength of the fluctuation in parameter estimates as a measure of statistical efficiency. Results show that efficiency and unbiasedness are closely related, such that in small samples, obtained estimation results might not just be biased, but also potentially different in size if the analysis is repeated with another sample from the same population. As the abovementioned studies did not evaluate the strength of estimation fluctuation, the ability to compare our results in this respect is limited.

### Recommendations for Researchers

This simulation study is meant to help researchers decide on sufficient sample sizes for a three-level longitudinal study with (assumed) population and sample characteristics, effects and models similar to the simulated conditions.

Given that the proposed model and data are comparable to the one examined in this simulation, researchers should consider at least 15 students each in 35 classes for stable fixed effects results, and more students when the analysis of small effects is considered important. If r.e. variances are of interest, additional level-3 units are required, particularly for higher-level effects, where more than 55 classrooms are needed.

If primarily estimation bias is of concern, researchers should consider sampling at least 15 students per classroom regardless of coefficient size, in order to equally decrease bias and fluctuation in estimation results. If significance tests or confidence intervals are used for interpretation, sufficient power for relatively large effects is reliably reached by sampling at least 15 classes with 15 students each. Smaller effects, however, require larger samples with 35 students in each class, which is a difficult task given that such large classrooms are the exception (see OECD, 2013 for worldwide average class sizes).

### Limitations and Future Prospects

Results of this case-study are naturally limited by the study design and sample characteristics of the illustrative study. Particularly, results and recommendations are specific to settings where researchers can assume comparable population and sample characteristics (e.g., distribution of variables, ICC values) and use comparable predictor and outcome variables with similar expected effect sizes. Additionally, recommendations are a result of the model fitted to the data [i.e., as in (9) to (11), including predictors on all levels, cross-level-interactions, random intercepts, and random slopes]. As a consequence, sample size recommendations are not ubiquitously generalizable. Especially if the model or data give reason to suspect lower statistical power due to smaller effect sizes or a more complex model than investigated in this study, the recommended samples sizes will most likely be too small. On the other hand, if the assumed power is higher, e.g., due to larger expected effect sizes, the recommendations presented might be conservative, and thus serve as an approximate upper bound for required sample sizes, as it is not necessary to increase sample sizes above the provided recommendations. In addition to that, we limited our analyses to data with balanced sample sizes, since in each sample, there is a fixed number of students per class, and students attend or fail to attend at a fixed number of measurement occasions. We therefore did not investigate how estimation quality might be different for unbalanced designs, for example in samples where classes differ with respect to the number of students. In practice, these unbalanced designs can occur either naturally due to the sampling process, or deliberately, e.g., by oversampling students in classes with higher expected attrition over time. Potentially, oversampling might prevent bias or loss of statistical power due to expected missingness, and the strength of the imbalance might further influence estimation quality on all levels. To assess the potential benefits and consequences of oversampling, future research might focus on comparing estimation quality of balanced samples to samples with unequal cluster sizes, but on average the same sample sizes per level as the balanced data.

Furthermore, we did not reduce the level-2 sample size to two students per class (dyadic data) due to the restricted number of admissible random effects at the higher level: data have to contain more units per cluster than random effects at this level (Kenny et al., 2006; Du and Wang, 2016). In order to scrutinize the extent of necessary simplification, we conducted additional analyses by drawing samples of two students per classroom (100 replications for each N_3_ condition without missing values) for reduced models, i.e., (a) a three-level model with random intercepts at levels two and three, but not random slopes, and (b) a two-level model with a random intercept, but no random slope and no consideration of the classroom level. For both reduced models, more than a third of analysis runs resulted in r.e. variance components being estimated as close or equal to zero, leading to a singular fit. In conclusion, sampling only two students per class would require a much simpler analysis model for successful estimation, which would make evaluations of estimation quality incomparable to the initial sample size conditions, and inferred sample size recommendations would not be based on the same underlying model specifications.

While the complexity of the statistical model and the specificity of the data and analysis conditions are meant to ensure practical relevance for the field, there are a variety of additional potential factors which influence the required sample size but were not investigated in this study. For example, since we based our parameter values on one illustrative study, the ICC values were not varied as an additional simulation condition. As the ICC is a direct representation of the (unexplained) pronouncedness of the multilevel structure and hence relevant if the estimation of random effects is of interest, varying ICC values in three-level models relevant for applied psychological research can provide a better understanding of the importance of each data level for accurate estimation results. To ensure practical relevance, future studies might generate data according to reported ICC values in the field, or alternatively base their simulations on a variety of sample studies depicting different estimated r.e. variance components. For longitudinal studies, measurement occasions can be varied to more clearly examine the importance of the level-1 sample size, i.e., the longitudinal component. In this regard, future studies should consider examining systematic dropout in order to provide recommendations for samples with systematic missing values, as researchers oftentimes are unsure how to handle such missing data (cf. Jeličić et al., 2009, for an overview). Other characteristics, such as multicollinearity and heteroscedasticity, should be further examined, as such characteristics are not uncommon in applied research, but not comprehensively studied in three-level models (in two-level models: cf. Korendijk et al., 2008, for heteroscedasticity; Shieh and Fouladi, 2003, for multicollinearity). In conclusion, analyses of a representative choice of study designs and settings might assist researchers in choosing the adequate sample sizes, sample size allocations, and variables, for a variety of research questions.

## Author Contributions

DK contributed by conducting extensive research on the topic of the manuscript, conducting statistical analyses, and writing the manuscript. FN contributed by providing expertise, supervision with regards to statistical analyses, and proofreading.

## Conflict of Interest Statement

The authors declare that the research was conducted in the absence of any commercial or financial relationships that could be construed as a potential conflict of interest.

## Footnotes

1 Following arguments by Bates (2006), the reliability of significance tests for coefficients of the MLA-model is controversial, as degrees of freedom cannot be conclusively determined. Hence, the lmer()-function does not calculate *p*-values. In order to provide an approximate measure for statistical significance, we calculated confidence intervals using the confint()-function for the lmer() model parameter estimates. Coverage and power results in this contribution hold only under the assumption that degrees of freedom are reliably estimated.

2 We henceforth refer to regression coefficients associated to predictions of intercepts using the name of the predictor (e.g., classtype refers to the level-3 regression coefficient), and to regression coefficients associated to predictions of slope parameters by the product of the variables (e.g., time × classtype).

## Abbreviations

COM: simulated condition indicating complete data without missing values
DrOP2: simulation condition indicating data with a dropout pattern such that 20% of level-2 units do not produce data for the last two measurement occasions
DrOP3: simulation condition indicating data with a dropout pattern such that 20% of level-2 units do not produce data for the last three measurement occasions
ES: effect size
ICC: intraclass correlation coefficient
MCAR: simulation condition indicating data with 20% of level-1 values (measurement occasions) missing completely at random
MLA: multilevel analysis
*Peb*: parameter estimation bias
