# Supplementary material for: The Influence of Sample Size on Parameter Estimates in Three-Level Random-Effects Models
Source: Front Psychol. 2019 May 21;10:1067. doi: 10.3389/fpsyg.2019.01067 (PMC6536630; doi:10.3389/fpsyg.2019.01067)
Supplement: Supplementary file 1 [file Table_1.docx]

| Table A.1.  *Parameter estimation bias (*peb*) of samples with missing value pattern MCAR for fixed effects.* | | | | | | | | | |
| --- | --- | --- | --- | --- | --- | --- | --- | --- | --- |
|  | intercept | aatimeaa | classtype | gender | influence | time× aatimeaa | classtype× aatimeaa | gender× aatimeaa | influence×  classtype |
| N_2_/ N_3_ | *peb* | *peb* | *peb* | *peb* | *peb* | *peb* | *peb* | *peb* | *peb* |
| 5/15 | .003 | .021 | -.119 | 1.149 | -.042 | -.021 | -.203 | -.538 | -.016 |
| 5/35 | .006 | .022 | -.148 | .444 | -.130 | -.018 | -.242 | -.432 | -.080 |
| 5/55 | .005 | .035 | -.163 | 1.148 | -.113 | -.004 | -.179 | -.536 | -.114 |
| 15/15 | -.003 | .010 | .041 | 1.211 | .030 | .014 | .096 | -.136 | .054 |
| 15/35 | -.003 | .011 | .033 | .979 | .033 | .024 | .148 | -.097 | .045 |
| 15/55 | -.004 | .014 | .041 | .965 | .029 | .020 | .097 | -.119 | .054 |
| 35/15 | <.001 | -.012 | .016 | -.246 | .002 | .005 | .088 | .094 | .010 |
| 35/35 | .002 | -.022 | .006 | -.873 | .006 | -.016 | .035 | .157 | -.009 |
| 35/55 | <.001 | -.010 | .016 | -.575 | .011 | -.009 | -.001 | .142 | .012 |
| *Notes*. N_2_=Number of students per class (level-2 sample size per group). N_3_=Number of classes (level-3 sample size). *MCAR* refers to the missing data pattern with 20% of reports missing at random measurement occasions. | | | | | | | | | |

| Table A.2.  *Parameter estimation bias (*peb*) of samples with missing value pattern DrOP2 for fixed effects.* | | | | | | | | | |
| --- | --- | --- | --- | --- | --- | --- | --- | --- | --- |
|  | intercept | aatimeaa | classtype | gender | influence | time× aatimeaa | classtype× aatimeaa | gender× aatimeaa | influence×  classtype |
| N_2_/ N_3_ | *peb* | *peb* | *peb* | *peb* | *peb* | *peb* | *peb* | *peb* | *peb* |
| 5/15 | .003 | .031 | -.116 | .839 | -.062 | -.003 | -.205 | -.508 | -.035 |
| 5/35 | .005 | .007 | -.165 | 1.548 | -.106 | -.031 | -.225 | -.486 | -.084 |
| 5/55 | .006 | .007 | -.160 | .808 | -.117 | -.035 | -.216 | -.408 | -.116 |
| 15/15 | -.004 | .006 | .052 | 1.318 | .027 | .004 | .071 | -.124 | .019 |
| 15/35 | -.003 | .003 | .044 | .778 | .030 | .014 | .088 | -.025 | .065 |
| 15/55 | -.003 | .006 | .039 | .820 | .035 | .010 | .087 | -.109 | .063 |
| 35/15 | <.001 | -.005 | .011 | -.608 | .008 | .006 | .040 | .192 | .002 |
| 35/35 | <.001 | -.008 | .012 | -.655 | .006 | .002 | .050 | .115 | .008 |
| 35/55 | <.001 | -.009 | .010 | -.309 | -.005 | <.001 | .031 | .130 | -.013 |
| *Notes*. N_2_=Number of students per class (level-2 sample size per group). N_3_=Number of classes (level-3 sample size). *DrOP2* refers to the missing data pattern with 20% of level-1 data missing at the last two measurements. | | | | | | | | | |

| Table A.3.  *Parameter estimation bias (*peb*) of samples with missing value pattern DrOP3 for fixed effects.* | | | | | | | | | |
| --- | --- | --- | --- | --- | --- | --- | --- | --- | --- |
|  | intercept | aatimeaa | classtype | gender | influence | time× aatimeaa | classtype× aatimeaa | gender× aatimeaa | influence×  classtype |
| N_2_/ N_3_ | *peb* | *peb* | *peb* | *peb* | *peb* | *peb* | *peb* | *peb* | *peb* |
| 5/15 | .003 | .045 | -.122 | .733 | -.056 | .009 | -.223 | -.541 | -.048 |
| 5/35 | .005 | .025 | -.172 | 1.502 | -.103 | -.013 | -.239 | -.510 | -.104 |
| 5/55 | .006 | .021 | -.165 | .724 | -.112 | -.022 | -.231 | -.432 | -.130 |
| 15/15 | -.004 | .010 | .053 | 1.311 | .025 | .011 | .080 | -.123 | .016 |
| 15/35 | -.003 | .006 | .046 | .771 | .031 | .019 | .095 | -.028 | .071 |
| 15/55 | -.003 | .009 | .040 | .792 | .034 | .014 | .094 | -.122 | .062 |
| 35/15 | <.001 | -.004 | .014 | -.587 | .012 | .008 | .042 | .201 | .012 |
| 35/35 | <.001 | -.008 | .014 | -.634 | .011 | .002 | .050 | .122 | .015 |
| 35/55 | <.001 | -.010 | .010 | -.275 | -.001 | -.001 | .030 | .140 | -.011 |
| *Notes*. N_2_=Number of students per class (level-2 sample size per group). N_3_=Number of classes (level-3 sample size). *DrOP3* refers to the missing data pattern with 20% of level-1 data missing at the last three measurements. | | | | | | | | | |

| Table A.4.  *Parameter estimation bias (*peb*) of samples with missing value pattern MCAR for random effects.* | | | | | |
| --- | --- | --- | --- | --- | --- |
|  | Level-3 intercept | Level-3 slope | Level-2 intercept | Level-2 slope | Level-1 residual |
| N_2_/ N_3_ | *peb* | *peb* | *peb* | *peb* | *peb* |
| 5/15 | 2.328 | .691 | -.079 | .195 | -.001 |
| 5/35 | .978 | .295 | -.062 | .090 | .006 |
| 5/55 | .580 | .233 | -.074 | .024 | .013 |
| 15/15 | .913 | .222 | -.027 | -.014 | -.004 |
| 15/35 | .546 | .123 | -.017 | -.022 | .001 |
| 15/55 | .394 | .119 | -.014 | -.011 | .001 |
| 35/15 | .110 | -.024 | .003 | -.006 | -.001 |
| 35/35 | -.058 | -.029 | .008 | .002 | -.002 |
| 55/55 | -.070 | -.028 | .012 | -.001 | -.002 |
| *Notes*. N_2_=Number of students per class (level-2 sample size per group). N_3_=Number of classes (level-3 sample size). *MCAR* refers to the missing data pattern with 20% of reports missing at random measurement occasions. | | | | | |

| Table A.5.  *Parameter estimation bias (*peb*) of samples with missing value pattern DrOP2 for random effects.* | | | | | |
| --- | --- | --- | --- | --- | --- |
|  | Level-3 intercept | Level-3 slope | Level-2 intercept | Level-2 slope | Level-1 residual |
| N_2_/ N_3_ | *peb* | *peb* | *peb* | *peb* | *peb* |
| 5/15 | 2.190 | .674 | -.073 | .103 | .001 |
| 5/35 | .849 | .347 | -.064 | .063 | .008 |
| 5/55 | .345 | .136 | -.065 | .030 | .011 |
| 15/15 | .856 | .301 | -.021 | -.041 | -.001 |
| 15/35 | .502 | .091 | -.011 | -.007 | -.001 |
| 15/55 | .372 | .043 | -.012 | -.026 | <.001 |
| 35/15 | .112 | -.020 | .012 | -.008 | -.001 |
| 35/35 | -.119 | -.042 | .013 | .005 | -.001 |
| 55/55 | -.124 | -.032 | .013 | .004 | -.001 |
| *Notes*. N_2_=Number of students per class (level-2 sample size per group). N_3_=Number of classes (level-3 sample size). *DrOP2* refers to the missing data pattern with 20% of level-1 data missing at the last two measurements. | | | | | |

| Table A.6.  *Parameter estimation bias (*peb*) of samples with missing value pattern DrOP3 for random effects.* | | | | | |
| --- | --- | --- | --- | --- | --- |
|  | Level-3 intercept | Level-3 slope | Level-2 intercept | Level-2 slope | Level-1 residual |
| N_2_/ N_3_ | *peb* | *peb* | *peb* | *peb* | *peb* |
| 5/15 | 2.254 | .693 | -.074 | .106 | .002 |
| 5/35 | .865 | .343 | -.064 | .060 | .010 |
| 5/55 | .391 | .138 | -.067 | .027 | .012 |
| 15/15 | .871 | .308 | -.022 | -.043 | -.001 |
| 15/35 | .481 | .107 | -.011 | -.014 | .001 |
| 15/55 | .346 | .056 | -.012 | -.031 | .001 |
| 35/15 | .119 | -.012 | .012 | -.011 | -.001 |
| 35/35 | -.105 | -.037 | .013 | .004 | -.001 |
| 55/55 | -.121 | -.029 | .015 | .004 | -.001 |
| *Notes*. N_2_=Number of students per class (level-2 sample size per group). N_3_=Number of classes (level-3 sample size). *DrOP3* refers to the missing data pattern with 20% of level-1 data missing at the last three measurements. | | | | | |

| Table A.7.  *Coverage rate of samples without missing values (COM).* | | | | | | | | | |
| --- | --- | --- | --- | --- | --- | --- | --- | --- | --- |
|  | intercept | aatimeaa | classtype | gender | influence | time× aatimeaa | classtype× aatimeaa | gender× aatimeaa | influence×  classtype |
| N_2_/ N_3_ | *cov* | *cov* | *cov* | *cov* | *cov* | *cov* | *cov* | *cov* | *cov* |
| 5/15 | 94.6 | 96 | 94.2 | 94 | 94.5 | 95.6 | 94.9 | 94.6 | 94.2 |
| 5/35 | 96.6 | 94.9 | 94.4 | 95.4 | 95.2 | 95.6 | 94.2 | 94.5 | 95.6 |
| 5/55 | 95.2 | 96 | 91 | 95.2 | 94.6 | 95.4 | 95.1 | 96.7 | 95 |
| 15/15 | 95.2 | 96.6 | 94.3 | 93.9 | 94.3 | 96.8 | 94.4 | 95.7 | 93.8 |
| 15/35 | 95.5 | 96 | 94.7 | 95.9 | 96 | 95.9 | 93.8 | 95.9 | 94.6 |
| 15/55 | 94.8 | 95.6 | 94.3 | 95.7 | 94.2 | 95.9 | 95.1 | 95.4 | 94.2 |
| 35/15 | 94.7 | 97.4 | 94.7 | 95.5 | 95.5 | 96.6 | 94 | 94.5 | 95.7 |
| 35/35 | 93.3 | 95.7 | 93.7 | 93.3 | 95.2 | 95.8 | 95.3 | 93.4 | 95 |
| 35/55 | 95.3 | 97.1 | 94.4 | 94.9 | 96.8 | 96.2 | 95.9 | 95.1 | 95.7 |
| *Notes*. *cov*=coverage rate in percent. N_2_=Number of students per class (level-2 sample size per group). N_3_=Number of classes (level-3 sample size). | | | | | | | | | |

| Table A.8.  *Coverage rate of samples with missing value pattern MCAR.* | | | | | | | | | |
| --- | --- | --- | --- | --- | --- | --- | --- | --- | --- |
|  | intercept | aatimeaa | classtype | gender | influence | time× aatimeaa | classtype× aatimeaa | gender× aatimeaa | influence×  classtype |
| N_2_/ N_3_ | *cov* | *cov* | *cov* | *cov* | *cov* | *cov* | *cov* | *cov* | *cov* |
| 5/15 | 94.7 | 95.3 | 95.7 | 94.1 | 94.5 | 94.6 | 95.4 | 94.2 | 94.5 |
| 5/35 | 95.8 | 94.3 | 95.4 | 95.8 | 95.6 | 94.4 | 94.4 | 93.9 | 95.1 |
| 5/55 | 96.2 | 96 | 91.5 | 95.6 | 94.8 | 95.1 | 94.9 | 94 | 95.4 |
| 15/15 | 94.5 | 94.6 | 95.5 | 95 | 93.8 | 94.6 | 94.1 | 95.9 | 93.6 |
| 15/35 | 93.7 | 96.2 | 95 | 95.4 | 94.1 | 94.9 | 94.9 | 95.8 | 93.7 |
| 15/55 | 95.9 | 96.1 | 94.2 | 95.7 | 95 | 94.4 | 95.3 | 95.9 | 94 |
| 35/15 | 95.8 | 95.9 | 96.4 | 95 | 95.8 | 96.1 | 94.3 | 94.8 | 93.3 |
| 35/35 | 93.6 | 95 | 94 | 94.1 | 95.6 | 95.5 | 94.9 | 94.7 | 95.1 |
| 35/55 | 95.8 | 96.8 | 94.6 | 96 | 96.5 | 96.4 | 95.1 | 93.7 | 95.8 |
| *Notes*. *cov*=coverage rate in percent. N_2_=Number of students per class (level-2 sample size per group). N_3_=Number of classes (level-3 sample size). *MCAR* refers to the missing data pattern with 20% of reports missing at random measurement occasions. | | | | | | | | | |

| Table A.9.  *Coverage rate of samples with missing value pattern DrOP2.* | | | | | | | | | |
| --- | --- | --- | --- | --- | --- | --- | --- | --- | --- |
|  | intercept | aatimeaa | classtype | gender | influence | time× aatimeaa | classtype× aatimeaa | gender× aatimeaa | influence×  classtype |
| N_2_/ N_3_ | *cov* | *cov* | *cov* | *cov* | *cov* | *cov* | *cov* | *cov* | *cov* |
| 5/15 | 93.7 | 95.6 | 94.4 | 93.4 | 94.7 | 95.5 | 94.6 | 94.3 | 94.7 |
| 5/35 | 95.9 | 94.6 | 94 | 95.8 | 95.3 | 94.9 | 93.7 | 95.2 | 95.4 |
| 5/55 | 95.4 | 96.4 | 92 | 96 | 94.5 | 95.3 | 94.7 | 94 | 95.4 |
| 15/15 | 95.1 | 96.1 | 94.7 | 95.4 | 95 | 94.9 | 93.7 | 96 | 94.1 |
| 15/35 | 94.1 | 95.4 | 93.8 | 95.6 | 94.7 | 96.1 | 93.3 | 96.2 | 93.5 |
| 15/55 | 95.1 | 95.8 | 94.1 | 95.9 | 94.1 | 95.8 | 95.3 | 94.8 | 94.4 |
| 35/15 | 95.1 | 96.6 | 95.2 | 94.8 | 97.2 | 96.5 | 94.9 | 93.1 | 95.9 |
| 35/35 | 95.2 | 97.2 | 94 | 95.2 | 95.9 | 96.6 | 94.2 | 94.3 | 95.3 |
| 35/55 | 96 | 97.3 | 95.1 | 95.3 | 96.1 | 95.7 | 95.5 | 93.7 | 94.4 |
| *Notes*. *cov*=coverage rate in percent. N_2_=Number of students per class (level-2 sample size per group). N_3_=Number of classes (level-3 sample size). *DrOP2* refers to the missing data pattern with 20% of level-1 data missing at the last two measurements. | | | | | | | | | |

| Table A.10.  *Coverage rate of samples with missing value pattern DrOP3.* | | | | | | | | | |
| --- | --- | --- | --- | --- | --- | --- | --- | --- | --- |
|  | intercept | aatimeaa | classtype | gender | influence | time× aatimeaa | classtype× aatimeaa | gender× aatimeaa | influence×  classtype |
| N_2_/ N_3_ | *cov* | *cov* | *cov* | *cov* | *cov* | *cov* | *cov* | *cov* | *cov* |
| 5/15 | 93.2 | 95.1 | 94.5 | 93.6 | 95 | 95.7 | 94.7 | 95 | 94.9 |
| 5/35 | 95.4 | 94.3 | 94.3 | 95.8 | 95.4 | 93.7 | 93.7 | 95 | 95.7 |
| 5/55 | 94.7 | 95.8 | 91.8 | 95.6 | 94.4 | 95.7 | 94.3 | 94.3 | 94.9 |
| 15/15 | 94.4 | 95.2 | 94.5 | 95.5 | 94.9 | 95.1 | 94.5 | 95.9 | 94.4 |
| 15/35 | 94.1 | 94.6 | 94.2 | 95.6 | 94.9 | 95.4 | 93.7 | 96.1 | 94.6 |
| 15/55 | 95.4 | 96.1 | 94.6 | 96 | 95.1 | 95.5 | 95.3 | 94.7 | 94.5 |
| 35/15 | 95.4 | 96.4 | 94.9 | 95 | 97.3 | 96.3 | 94.9 | 93 | 96.5 |
| 35/35 | 95.4 | 96.8 | 93.9 | 94.7 | 95.8 | 96.6 | 94.6 | 94.4 | 95.5 |
| 35/55 | 95.8 | 96.5 | 94.9 | 95.2 | 95.9 | 95 | 95.9 | 93.5 | 94.5 |
| *Notes*. *cov*=coverage rate in percent. N_2_=Number of students per class (level-2 sample size per group). N_3_=Number of classes (level-3 sample size). *DrOP3* refers to the missing data pattern with 20% of level-1 data missing at the last three measurements. | | | | | | | | | |

| Table A.11.  *Power results for variables classtype, influence, and their interaction effect.* | | | | | | | | | | | | | | | | | | |
| --- | --- | --- | --- | --- | --- | --- | --- | --- | --- | --- | --- | --- | --- | --- | --- | --- | --- | --- |
|  | classtype | | | |  | | influence | | | |  | | influence×classtype | | | |  | |
| N_2_/ N_3_ | COM | MCAR | DrOP2 | DrOP3 | |  | COM | MCAR | DrOP2 | DrOP3 | |  | COM | MCAR | DrOP2 | DrOP3 | |  |
| 5/15 | 34.5 | 30.1 | 33.4 | 32.7 | |  | 22.5 | 16.6 | 19.2 | 19.0 | |  | 12.7 | 11.7 | 11.7 | 11.5 | |  |
| 5/35 | 67.0 | 60.2 | 64.6 | 62.8 | |  | 41.3 | 31.2 | 37.2 | 34.9 | |  | 20.9 | 17.9 | 18.0 | 16.5 | |  |
| 5/55 | 84.9 | 77.4 | 83.3 | 83.2 | |  | 56.5 | 45.2 | 50.8 | 49.3 | |  | 29.4 | 25.0 | 27.5 | 25.4 | |  |
| 15/15 | 81.4 | 76.6 | 82.0 | 81.8 | |  | 58.1 | 51.2 | 55.9 | 54.5 | |  | 32.4 | 25.9 | 28.1 | 27.0 | |  |
| 15/35 | 99.6 | 99.0 | 99.4 | 99.5 | |  | 92.4 | 82.1 | 88.3 | 86.5 | |  | 65.7 | 53.7 | 61.0 | 61.0 | |  |
| 15/55 | 100 | 100 | 100 | 100 | |  | 97.8 | 96.2 | 98.4 | 98.1 | |  | 83.7 | 74.6 | 82.1 | 79.9 | |  |
| 35/15 | 97.8 | 97.6 | 98.0 | 98.0 | |  | 88.0 | 79.3 | 84.4 | 83.1 | |  | 58.7 | 51.4 | 55.0 | 53.6 | |  |
| 35/35 | 100 | 100 | 100 | 100 | |  | 99.8 | 99.1 | 99.6 | 99.7 | |  | 93.8 | 86.1 | 91.2 | 90.3 | |  |
| 35/55 | 100 | 100 | 100 | 100 | |  | 100 | 100 | 100 | 100 | |  | 99.6 | 97.8 | 98.5 | 97.4 | |  |
| *Notes*. N_2_=Number of students per class (level-2 sample size per group). N_3_=Number of classes (level-3 sample size). Values describe the percentage of samples where the confidence interval of the parameter estimate does not include zero. COM to DrOP3 correspond to missing value patterns described in the *Materials and* *Methods* section and Table 3. | | | | | | | | | | | | | | | | | | |

| Table A.12.  *Power results for the linear and quadratic time effect.* | | | | | | | | | | |
| --- | --- | --- | --- | --- | --- | --- | --- | --- | --- | --- |
|  | •••••••••••••••••••••••time•••••••••••••••••••••••• | | | | | ••••••••••••••••••••time×time•••••••••••••••••••• | | | | |
| N_2_/N_3_ | COM | MCAR | DrOP2 | DrOP3 |  | COM | MCAR | DrOP2 | DrOP3 |  |
| 5/15 | 43.0 | 32.5 | 38.5 | 36.3 |  | 30.7 | 22.5 | 27.7 | 26.7 |  |
| 5/35 | 75.4 | 65.0 | 72.4 | 71.0 |  | 53.6 | 45.9 | 51.3 | 49.7 |  |
| 5/55 | 92.5 | 86.2 | 90.5 | 88.3 |  | 77.2 | 65.6 | 70.9 | 68.8 |  |
| 15/15 | 83.8 | 72.8 | 79.8 | 77.5 |  | 69.8 | 59.0 | 65.2 | 63.6 |  |
| 15/35 | 99.5 | 98.0 | 98.7 | 98.3 |  | 97.7 | 92.7 | 96.0 | 95.1 |  |
| 15/55 | 100 | 100 | 100 | 100 |  | 99.8 | 98.7 | 99.6 | 99.3 |  |
| 35/15 | 98.5 | 96.0 | 98.6 | 98.0 |  | 98.0 | 92.2 | 96.1 | 94.9 |  |
| 35/35 | 100 | 100 | 100 | 100 |  | 100 | 100 | 100 | 100 |  |
| 35/55 | 100 | 100 | 100 | 100 |  | 100 | 100 | 100 | 100 |  |
| *Notes*. N_2_=Number of students per class (level-2 sample size per group). N_3_=Number of classes (level-3 sample size). Values describe the percentage of samples where the confidence interval of the parameter estimate does not include zero. COM to DrOP3 correspond to missing value patterns described in the *Materials and Methods* section and Table 6. | | | | | | | | | | |
