# Supplementary material for: The Influence of Sample Size on Parameter Estimates in Three-Level Random-Effects Models
Source: Front Psychol. 2019 May 21;10:1067. doi: 10.3389/fpsyg.2019.01067 (PMC6536630; doi:10.3389/fpsyg.2019.01067)
Supplement: Supplementary file 2 [file Table_2.DOCX]

*Figure A.1* Parameter estimation bias (*peb*) for main fixed effects estimates, in relation to the standard deviation of their estimated parameter values over 1000 simulation runs for each sample size condition. Shades differentiate between numbers of students per class (N_2_), shapes differentiate between numbers of classrooms per sample (N_3_). As results are very similar between samples with and without missing values, plots do not differentiate between missing value patterns.

*Figure A.2* Parameter estimation bias (*peb*) for interaction effects estimates, in relation to the standard deviation of their estimated parameter values over 1000 simulation runs for each sample size condition. Shades differentiate between numbers of students per class (N_2_), shapes differentiate between numbers of classrooms per sample (N_3_). As results are very similar between samples with and without missing values, plots do not differentiate between missing value patterns.

*Figure A.3*. Parameter estimation bias (*peb*) for random effects estimates, in relation to the standard deviation of their estimated parameter values over 1000 simulation runs for each sample size condition. Shades differentiate between numbers of students per class (N_2_), shapes differentiate between numbers of classrooms per sample (N_3_). As results are very similar between samples with and without missing values, plots do not differentiate between missing value patterns.
