# Supplementary figures and images for: The Influence of Sample Size on Parameter Estimates in Three-Level Random-Effects Models
Source: Front Psychol. 2019 May 21;10:1067. doi: 10.3389/fpsyg.2019.01067 (PMC6536630; doi:10.3389/fpsyg.2019.01067)

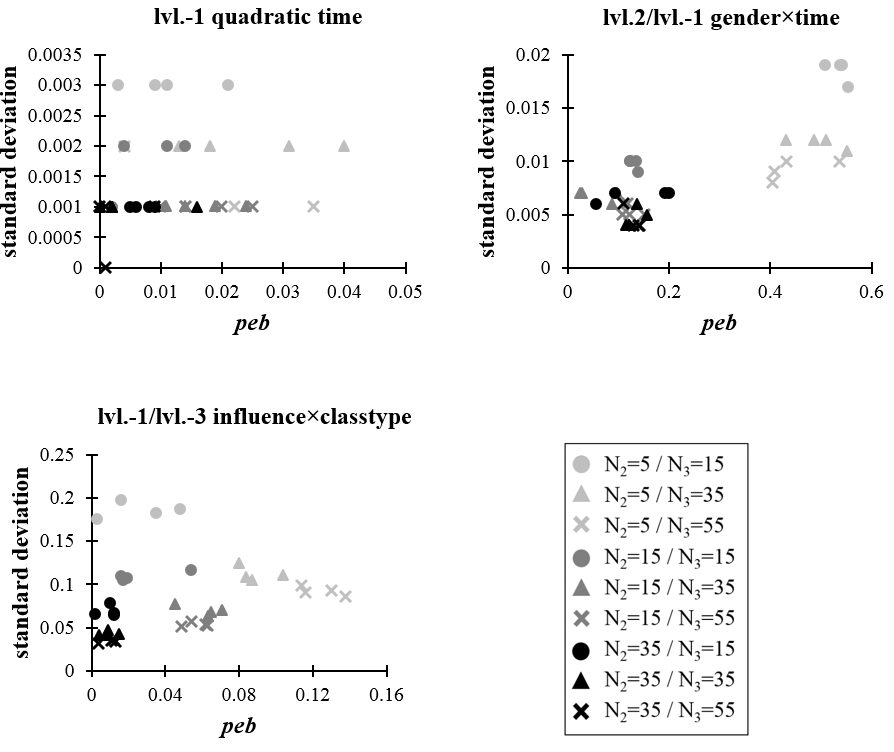

Supplement: Supplementary file 4 [file Image_2.TIFF]
